# An Animal‐Free Patient‐Derived Tissue‐Mimetic Biochip Model of the Human Synovial Membrane for Human‐Relevant Osteoarthritis Research

**DOI:** 10.1002/adhm.202404799

**Published:** 2025-06-29

**Authors:** Eva I. Reihs, Alexander Stoegner, Mateo G. Vasconez Martínez, Markus M. Schreiner, Melanie Cezanne, Ruth Gruebl‐Barabas, Bettina Rodriguez‐Molina, Juergen Alphonsus, Silvia Hayer, Richard Lass, Iris Gerner, Florien Jenner, Wolfgang Holnthoner, Stefan Toegel, Peter Ertl, Hans P. Kiener, Reinhard Windhager, Mario Rothbauer

**Affiliations:** ^1^ Department of Orthopedics and Trauma Surgery, Karl Chiari Lab for Orthopaedic Biology Medical University of Vienna Waehringer Guertel 18–20 Vienna 1090 Austria; ^2^ Department of Orthopedics and Trauma Surgery Division of Orthopedics Medical University of Vienna Waehringer Guertel 18–20 Vienna 1090 Austria; ^3^ Institute of Applied Synthetic Chemistry Institute of Applied Synthetic Chemistry CellChipGroup Technische Universitaet Wien (TUW) Getreidemarkt, 9/163 Vienna 1060 Austria; ^4^ Division of Rheumatology Department of Medicine III Medical University of Vienna Waehringer Guertel 18–20 Vienna 1090 Austria; ^5^ Veterinary Tissue Engineering and Regenerative Medicine Vienna (VETERM) Equine Surgery Clinics University of Veterinary Medicine Vienna Veterinärplatz 1 Vienna 1210 Austria; ^6^ AUVA Research Centre Ludwig Boltzmann Institute for Experimental and Clinical Traumatology Vienna 1200 Austria; ^7^ Ludwig Boltzmann Institute of Arthritis and Rehabilitation Vienna 1090 Austria

**Keywords:** disease modeling, non‐animal methods, organ‐on‐a‐chip, osteoarthritis, synovium

## Abstract

Current synovial models fail to capture human‐relevant OA traits. This study develops a fully humanized, animal‐free synovial membrane model, mimicking OA synovial structure and molecular profile. Protocols for rheumatoid synovial micromasses are adapted for 3D biochip cultures of OA synoviocytes using TISSEEL fibrin and ELAREM lysate. Cell activity, mRNA expression, and structural changes are evaluated under varying hydrogel stiffness and cytokine exposure, with results compared to human OA and animal (equine and murine) synovial tissues. The animal‐free biochip protocols replicate synovial architecture successfully. Improved gene expression of reticular collagen III (COL3A1) is achieved with 50 mg mL^−1^ fibrinogen and 1% hPL. A 50 pg mL^−1^ TNF‐α and IL‐1β stimulus induced a pro‐fibrotic phenotype (COL1A1, COL3A1) distinct from the inflammatory response triggered by ng/mL dosages (IL6, MMP1, MMP3, and MMP13, vs the pg/mL model). The clinical relevance of the patient‐relevant OA synovial model is underscored by significant Yap1 overexpression, reflecting synovial hyperplasia from cell activation and inflammation. Yap1 distribution, as a biomarker (ctrl vs kOA tissue), is best replicated in the low‐dose pg/ml‐treated model. The tissue‐mimetic biochips provide a human‐relevant OA study platform offering patient‐relevant molecular insights into the structure‐function relationships of osteoarthritic synovial tissues while eliminating animal‐derived materials.

## Graphical abstract

Reihs et al. present the development of a humanized, animal‐free synovial membrane model for osteoarthritis. Methods include 3D biochip cultures of human‐primary OA synoviocytes with matrix and serum surrogates. Results show replication of synovial architecture and increased Yap1 expression. Conclusions highlight the potential of these biochips for patient‐relevant molecular OA studies.

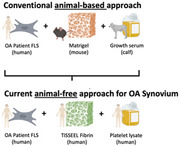

## 1 Introduction

Recent regulatory changes, increasing political pressures, social scrutiny, and heightened public awareness have fostered the development of alternative methods to refine, reduce, and replace animal experiments (3Rs).^[^ ^1^ ^]^ To minimize the use of animals and reduce their suffering in scientific research, various initiatives have been launched over the years including the establishment of ECVAM in 1991, the implementation of the REACH guideline in 2006, and Directive 2010/63/EU, all intended to establish standardized “non‐animal methods” (NAMs) for basic and applied biomedical and pharmaceutical sciences. A key paradigm shift occurred with the passage of the 2022 US legislation allowing pharmaceutical companies to apply for FDA approval using NAMs during pre‐clinical evaluations instead of animal testing.^[^ ^2^ ^]^ Among these developments, organ‐on‐a‐chip technologies play a crucial role, as they can provide more reproducible, reliable, and accurate representations of human physiological processes than animal models.^[^ ^3^ ^]^ Despite continuous technological advancements that incorporate tissue‐mimetic niches and embedded sensors,^[^ ^4^, ^5^ ^]^ human tissue in vitro models still rely on animal‐based products, including animal serum, cell culture products, scaffolding hydrogels, and cytokines. Although alternatives such as recombinant proteins and human‐derived matrices are being developed,^[^ ^6^, ^7^, ^8^ ^]^ the persistent and widespread use of animal‐derived serum supplements remains the main obstacle to eliminating animal products in biomedical research.

In this work, we set out to establish, for the first time, an authentic, entirely animal‐product‐free organ‐on‐a‐chip system capable of emulating human musculoskeletal tissue architecture while simulating the dynamic (patho)physiological responses of the synovial joint capsule. Notably, osteoarthritis (OA) remains one of the most common progressive musculoskeletal diseases affecting diarthrodial synovial joints, with the highest prevalence in knee joints (kOA),^[^ ^9^, ^10^ ^]^ yet it remains untreated. A variety of in vitro models, which still rely on animal products in their culturing methods, including explant cultures, pellet cultures,^[^ ^11^, ^12^ ^]^ micromass organoids, and microfluidic 3D models have been used in researching arthritic diseases across various tissues.^[^ ^13^, ^14^, ^15^, ^16^ ^]^ Despite the recent introduction of synovial joint‐on‐a‐chip systems,^[^ ^14^, ^15^, ^17^, ^18^, ^19^, ^20^ ^]^ most synovial disease models have primarily focused on rheumatoid arthritis research, and the role of the synovial membrane in osteoarthritis has been largely overlooked.^[^ ^21^, ^22^, ^23^, ^24^ ^]^

To advance human‐relevant OA disease modeling, we developed an animal‐ and animal‐product‐free culture protocol for the reliable generation of human synovial organoids (see Figure S1, Supporting Information for detailed protocol steps). An important aspect of our study design was the incorporation of various diseased donor origins using patient‐derived fibroblast‐like synovial cells, allowing us to account for disease variability, which was reflected in different cellular responses regarding structure and activity. Standard animal‐derived culture strategies (i.e., Matrigel, FCS serum supplement, Trypsin‐EDTA solution, and bovine serum albumin (BSA) carrier) were replaced with suitable human‐based surrogates (i.e., TISSEEL fibrin hydrogel, ELAREM human platelet lysate (hPL), TrypLE express solution and human serum albumin (HAS) as a carrier). Additional cell culture optimizations included adjusting hPL concentrations, ascorbic acid (AA) supplementation, and fine‐tuning the concentrations of fibrin hydrogel constituents (i.e., fibrinogen and thrombin). We then assessed the robustness and authenticity of our animal‐product‐free human synovium model through basal gene expression analysis of matrix and disease markers relevant to OA by quantitative real‐time polymerase chain reaction (qPCR) analysis, as well as the histological evaluation of synovial organoid architecture (i.e., lining and sublining characteristics), cellularity, and tissue‐specific markers and matrix components (i.e., collagen type I, collagen type III, lubricin). Moreover, genetic stability and patient variability were also investigated for animal‐free cultivation protocols. As a practical application, untreated synovial organoids, which were considered “healthy” controls, are stimulated using a cocktail of pro‐inflammatory cytokines (interleukin 1 beta (IL‐1β) and tumor necrosis factor‐alpha (TNF‐α) at levels found in OA patient synovial fluids (≈50 pg mL^−1^ range) and using a higher concentration of 50 ng mL^−1^, which is typically employed to initiate OA phenotypes in published in vitro synovial cell cultures.^[^ ^25^, ^26^ ^]^ In the final set of experiments, distinctive changes of synovia in OA are investigated using gene expression analyses of OA biomarkers. At the same time, we conducted a comparative structural evaluation of synovial organoid lining thickening against non‐OA as well as diseased human synovial tissues from OA patient origins.

## 2 Results and Discussion

### 2.1 Establishment of an Entirely Human‐Based Approach for Organ‐on‐a‐Chip Technology, Combining Human Cells of Disease Origin with Animal‐Free Products for Authentic Organoid Structure and Function

**Figure 1:**
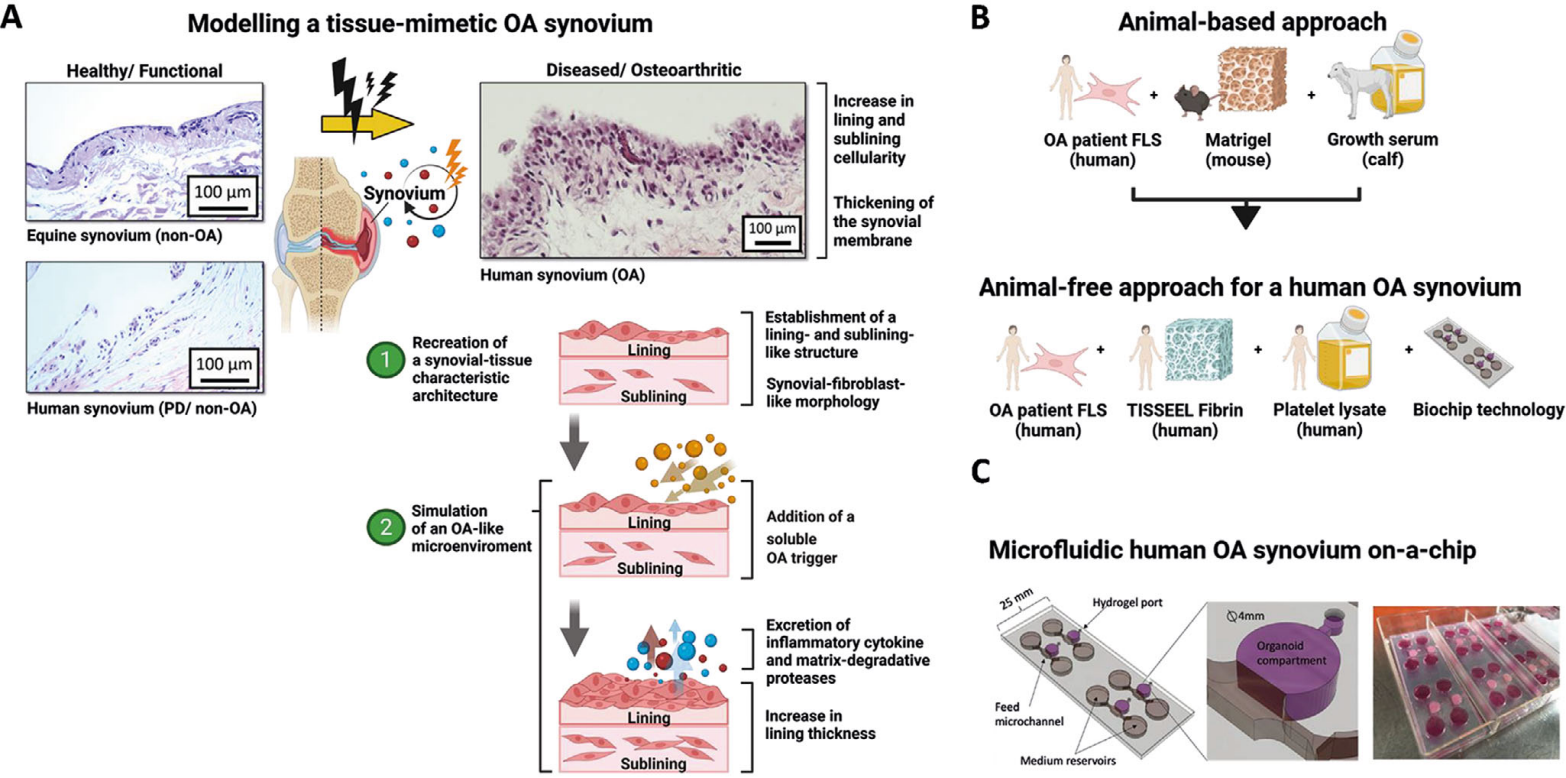
(A) Schematic overview of key steps for tissue‐mimetic modeling of osteoarthritic (OA) synovium, illustrating (1) the recreation of synovial tissue architecture and (2) the simulation of an OA‐like microenvironment. Histological sections depict structural features of OA‐affected synovium from knee joint tissue of OA patients (right), compared to representative healthy reference tissue: an equine non‐OA synovial section and a human non‐OA synovium derived from polydactyly (PD) surgery (left). These non‐OA samples serve as models for physiological baseline architecture and stromal organization. (B) Basic conceptual framework for transitioning animal‐based synovial organoids to a novel, non‐animal model for synovial disease using human OA primary synoviocytes, commercial TISSEEL fibrin hydrogel, and commercial ELAREM™ hPL growth supplement. (C) Photograph and schematic of biochip slides featuring four individual synovial organoid cultures, designed to fit in standard Quadriperm™ plates.

From an anatomical perspective, the healthy synovium is a highly organized structure between the joint cavity and the fibrous joint capsule. It consists of two distinct layers: a lining layer (intima synovialis), composed of densely packed cells typically arranged in a few cell layers, and a loosely organized sublining layer (subintima) containing extracellular matrix, scattered fibroblasts, macrophages, nerves, blood vessels, and lymphatics. **Figure** **1**A illustrates how the synovial lining layer is altered in osteoarthritis compared to healthy synovium, characterized by thickening of the lining layer (synovial hyperplasia). The creation of in vitro human musculoskeletal tissue models including synovial models, still relies on animal‐derived products such as animal‐derived serum and matrix products, as indicated in Figure 1B. The primary challenge in generating an animal‐product‐free human synovium‐on‐a‐chip system is adapting standard cell isolation and culture protocols to 10% hPL, achieving comparable outgrowth results to 10% fetal calf serum. Over 4–6 weeks, cell densities and cell morphology were investigated, exhibiting properties associated with fibroblast‐like synoviocyte populations (Figure S2, Supporting Information). Additionally, porcine pancreatic Trypsin for cell detachment was replaced with animal‐free recombinant TrypLE solution featuring similar cell detachment characteristics without altering the cell morphology and proliferation capacity of primary fibroblast‐like synoviocytes (Figure S2A, Supporting Information). Moreover, the development of cellular protrusions that are visible as an expansion on the 2D surface was defined as another key parameter of fibroblast adaptation and characterized in response to animal‐product‐free hPL and animal‐based FCS (see Figure S2B, Supporting Information). The results demonstrated that both growth supplements hPL and FCS supported cellular elongation, as documented by imaging on day 3 post‐cell‐seeding. An average cell length of 81 ± 26 µm was observed in synovial fibroblast supplemented with 1% hPL, compared to 57 ± 28 µm in the presence of 10% hPL. Next, the commonly used mouse‐derived Matrigel hydrogel matrix was replaced with clinical‐grade TISSEEL hydrogel, a natural hydrogel type that can be readily remodeled by primary cells during organoid maturation within the microfluidic biochip system. To test the animal‐product‐free synovial organ model, a plasma‐treated, UV‐sterilized, in‐house‐built biochip system was used (see Figure 1C). Each biochip consisted of four individual units containing several hydrogel compartments, each housing a single synovial organoid, connected to two medium reservoirs via a feed microchannel.

**Figure 2:**
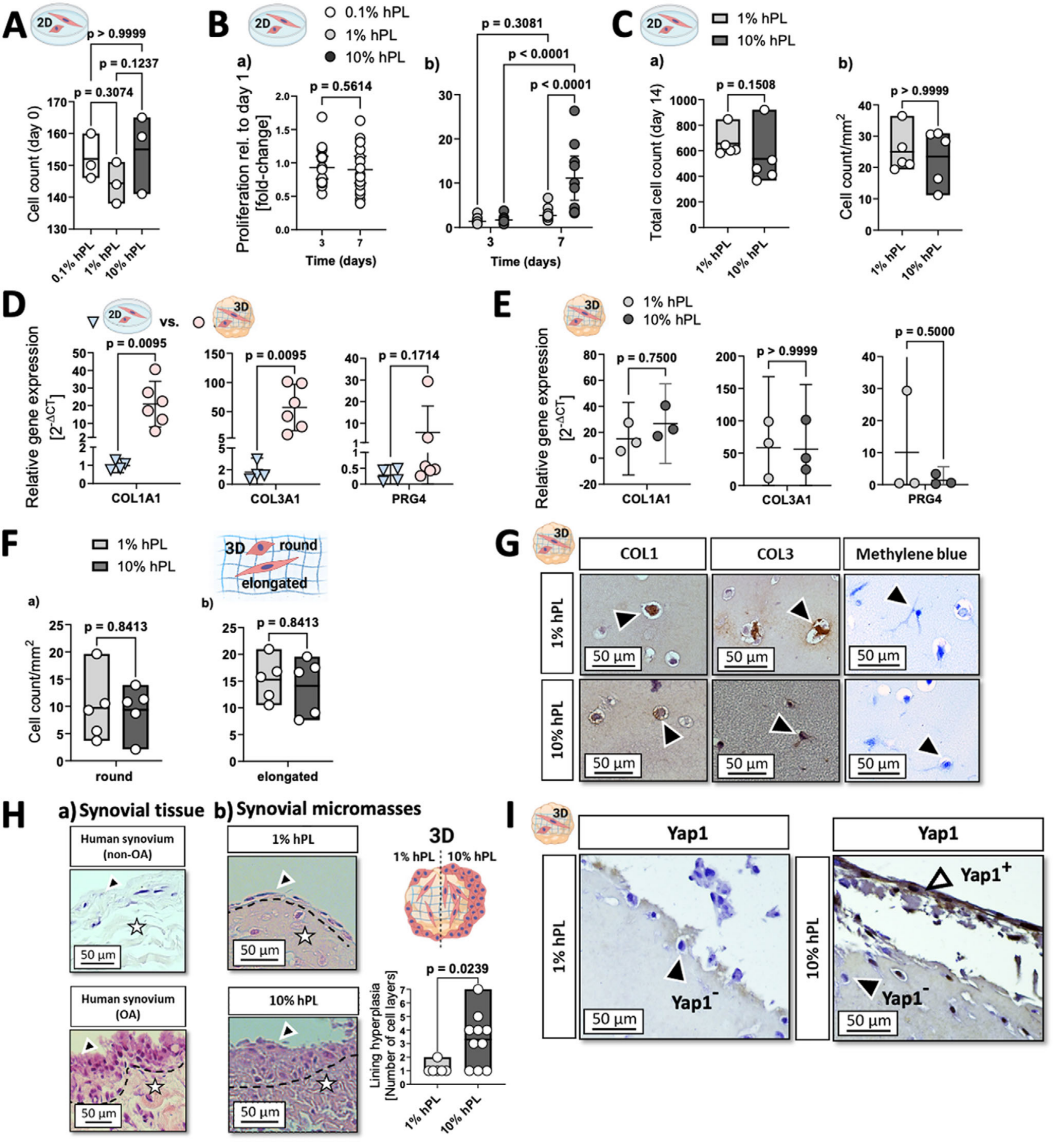
(A,B) Impact of hPL concentration on (A) the initial cell density attached on a cell culture plastic surface (n = 3), and (B a and b) time‐dependent proliferation of OA patient tissue‐derived synovial fibroblasts isolated and maintained with non‐animal products over a 7‐day culture period (n = 15). (C,D) Impact of hPL concentration on (C) total cell count a) and cellularity b) of synovial fibroblasts after 14 days of culture using non‐animal protocols (n = 5). (D) Comparison of gene expression of COL1A1, COL3A1, and PRG4 mRNA for primary OA patient‐derived synovial fibroblasts maintained with non‐animal protocols as 2D monolayers or 3D biochip organoids after 14 days of maturation (n = 4‐6). (E) Impact of hPL concentration on gene expression of COL1A1, COL3A1, and PRG4 genes for 3D biochip organoids matured over 14 days of maturation (n = 3) as well as (F) fibroblast‐like a) and round b) cell morphologies of seeded synovial fibroblasts (n = 5). (G) Brightfield images of immunohistological and histochemical analyses of synovial biochip organoids at day 14 post‐seeding for collagen type I and III, as well as DMMB staining for 1% and 10% hPL supplementation. (H) Brightfield images of a) lining and sublining regions of H&E‐stained non‐OA (cadaveric origin) and OA patient synovia in comparison to b) day 14 synovial biochip organoids matured in 1% (n = 6) and 10% hPL‐containing (n = 10) culture medium, as well as the analysis of synovial lining layer formation in corresponding biochips, and (I) respective images of synovial organoids using DAB‐immuno‐staining directed against Yap1. Data are either expressed as min‐to‐max mean % values + mean (A, C, F, and H) or as mean with 95% CI (B, D, and E). Statistical analyses include multiple Wilcoxon matched‐pairs analysis (B a, and E) or Mann Whitney U test (C a and b, D, F a and b, and H). For multiple comparison analyses, Friedman test (A), Kruskal‐Wallis with Dunn's multiple comparisons post hoc test, and 2‐way ANOVA (B b) were applied. Primary antibodies used included recombinant anti‐human Yap1 (1:50, Abcam), collagen type I (1:50), and collagen type III (1:50). The given sample number (n) always refers to biological replicates/different patient samples.

Before the establishment of animal‐product‐free on‐chip synovial organoids, the impact of human platelet lysate (hPL) as serum replacement on cell proliferation and physiology was investigated in subsequent experiments. In a comparative study, we explored fundamental properties of fibroblast‐like synoviocytes, focusing on metabolic activity, cell proliferation, and baseline analysis of gene expression related to matrix‐biosynthesis, matrix‐degradation/remodeling, pro‐inflammatory cytokines, and modulatory factors such as yes‐associated protein 1 (Yap1). As shown in **Figure** **2**A, the initial number of attached cells on day 0 was comparable regardless of hPL concentration in the culture medium (0.1% vs 1% hPL, p = 0.3074, 1% vs 10% hPL, p = 0.1237, and 0.1% vs 10% hPL, p>0.9999), with counts ranging from 135 to 165 cells per 0.875 cm^2^ when seeded at a cell density of ≈1443 cells cm^−2^. However, after seven days of 2D monolayer culture in the presence of increasing concentrations of hPL (from 0.1% to 10%), PrestoBlue™ staining (Figure S3A, Supporting Information) indicated an upregulation of intracellular esterase activity of primary fibroblast‐like synoviocytes (low passage number, p2 to 3) compared to the enzymatic activity on day 1. Interestingly, hPL supplementation of 0.1% resulted in low cell metabolism, showing a linear relationship with increasing cell numbers (R^2^ 0.998), peaking at 4145 ± 2654 AU for 20000 initially seeded cells. In contrast, 10% hPL showed a sigmoidal relationship between the increase in cell number and metabolic rates, peaking at 46 392 ± 2153 AU with a plateau phase starting at 10000 cells, most likely attributed to overcrowding on the culture surface. The 1% hPL supplementation demonstrated a linear relationship (R^2^ 0.90), with a peak metabolic activity at 12 962 ± 4140 AU. Furthermore, variability between patient‐derived cultures (n = 11) listed in Table S1 (Supporting Information) confirms that 1% hPL supplementation yielded the most reproducible results between the individual patient‐derived synoviocyte cultures with an initial seeding density of, i.e., 5000 cells and a relative standard deviation (RSD) of 44 ± 10%. In comparison, 10% and 0.1% supplementation resulted in RSDs of 51 ± 38% and 62 ± 28%, respectively. Similarly, Figure 2B showed a lack of cell proliferation during the 7‐day culture period, with 0.1% hPL inhibiting cell growth between day 3 and day 7 (day 7 vs day 3, p = 0.5614, 0.9290 mean fold‐change (fc) of day 3 vs day 1 and 0.8996 mean fc of day 7 vs day 1). In contrast, 1% and 10% hPL formulations showed either a strong or significant increase in cell proliferation by day 7 post‐seeding (vs day 3, p = 0.3081 for 1% hPL, and p<0.0001 for 10% hPL), with approximately two and tenfold mean increase of cell numbers relative to day 1, respectively. As a result, 0.1% hPL supplementation was not further investigated due to its lack of impact on cell activity and proliferation.

Next, we investigated the effects of 1% and 10% hPL supplementation on the formation of 3D synovial organoids generated using our synovium‐on‐a‐chip platform. Initial cell proliferation and shape analysis using H&E staining of histological sections of the center of the organoids confirmed comparable cell numbers ≈ 500–600 cells (see Figure 2C a p = 0.1508 and b p>0.9999), as well as consistent synoviocyte numbers with donor‐specific morphological variations after a 14‐day culture period in a 3D environment (see Figure 2F a and b p = 0.8413). A comparative study (Figure 2D) of mRNA expression of synovial collagens and lubricin confirmed that synoviocytes cultured in a microfluidic 3D environment upregulated collagen type I and III mRNA levels by ≈20‐ and 50‐fold (significance level for both markers: p = 0.0095 for 3D vs 2D), respectively. Interestingly, lubricin expression levels showed high variability in comparison to synoviocytes cultured in 2D (p = 0.1714), with a trend toward an overall increasing mean fold‐change of ≈5 (3D vs 2D). Overall, mRNA expression in the 3D organoids cultured with hPL showed no significant changes or upward trends, as seen in previous 2D/3D comparisons (Figure 2E), with similar collagen and lubricin expression at both 1% and 10% hPL supplementation. Additionally, collagen matrix expression in the presence of 1% and 10% hPL was confirmed by immunohistochemical and DMMB (1,9‐dimethylmethylene blue) staining, as shown in Figure 2G with visualizations of Collagens type I and III, and sulfated glycosaminoglycans (sGAGs). Results of structural organoid analysis (Figure 2H) further indicated that 1% hPL lysate produced synovia of a healthier synovial‐architectural phenotype (see Figure 2H a) with a lining layer thickness of one or two cells. In contrast, 10% supplementation resulted in the tendency to form a thicker lining (>3 cells), characteristic of arthritic features seen in rheumatoid arthritis or late‐state osteoarthritis.^[^ ^23^, ^27^, ^28^ ^]^ The quantitative analysis of pathobiological lining formation (Figure 2H b) revealed increased cellularity of the intimal‐lining layer, according to Krenn's scoring, with higher serum levels of 10% (10% vs 1% hPL, p = 0.0239). Furthermore, Figure 2I shows that Yap1 upregulation was observed for synovial organoids maintained in a 10% hPL‐supplemented culture medium, indicating a pathobiological response. Based on unpublished data generated during a previous study using biopsies of rheumatoid arthritis (RA) patients, a comparative analysis (Figure S3B, Supporting Information), revealed that rheumatoid synoviocyte cultures became hyperactive and exhibited unexpected architectural changes and signs of condensation when exposed to increasing hPL concentrations. These changes in the lining and sublining architecture occurred progressively with rising hPL concentrations, with the most pronounced substructural changes for 10% hPL. In other words, rRA‐derived micromasses exhibited drastic changes in sublining architecture, including unwanted thickening of fibroblast network structures and the formation of dense cell clusters throughout the sublining, with secondary synovial structures forming in the lining layers. Interestingly, fibroblast‐like synoviocyte cultures, obtained from osteoarthritic patients, did not show the same over‐stimulated response to hPL. These differences in responsiveness and activation status empathize with the critical role of disease origin in developing animal‐free protocols, as they influence tissue‐mimetic response. This simple observation highlights the necessity of thoroughly investigating the disease origin of primary cells when transitioning from existing animal‐based protocols to non‐animal methods.

### 2.2 Ascorbic Acid Supplementation Enhances Matrix Biosynthesis of Human Synovial Biochip Organoids while Dampening Pro‐Inflammatory Gene Signatures

**Figure 3:**
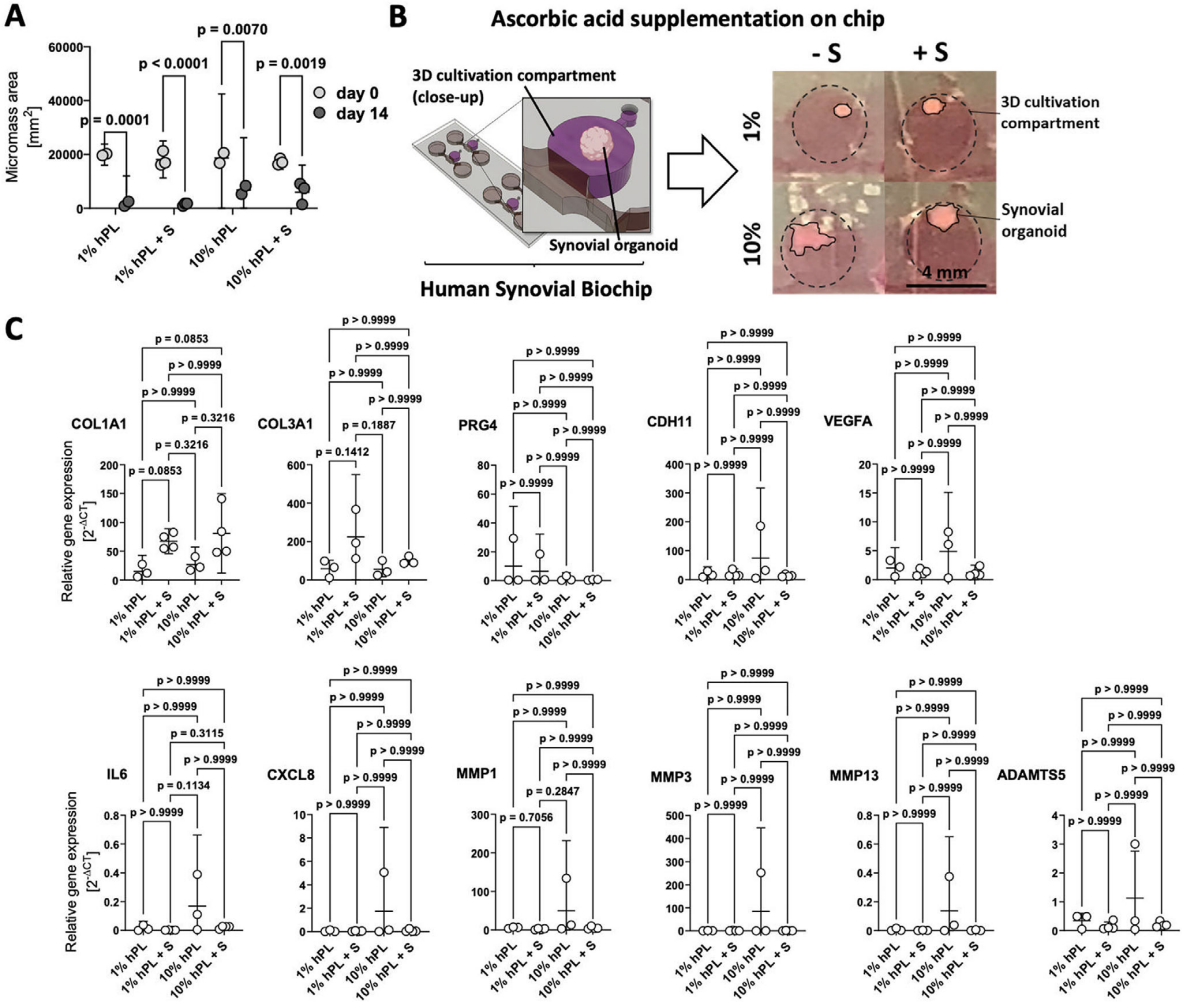
(A,B) Impact of ascorbic acid supplementation (+S; 100 µm) on the condensation dynamics of synovial biochip organoids (n = 3) shown in (A) as comparison between day 3 and day 14 post‐seeding as well as images (B) of day 14 biochip‐cultured constructs. (C) Comparison of gene expression of COL1A1, COL3A1, PRG4, CDH11, VEGFA, IL6, CXCL8, MMP1, MMP3, MMP13 and ADAMTS5 for primary OA patient‐derived synovial fibroblasts maintained with non‐animal protocols in the presence and absence of 100 µm ascorbic acid after 14 days of maturation (n = 3‐4), data are expressed as mean with 95% CI with 2 ‐way ANOVA (A) and Kruskal‐Wallis analysis (C). The given sample number (n) always refers to biological replicates/different patient samples.

Ascorbic acid (AA), a necessary cofactor for synovial matrix collagen synthesis, also contributes to the reduction of pro‐inflammatory cytokines and enhances the expression of protective factors involved in synovium healing. Consequently, the effect of 100 µm AA on organoid generation was investigated in subsequent experiments. In these experiments, the surface area of synovial organoids was continuously monitored between days 1 and 14 for culture groups that received 1% or 10% hPL in the absence (‐S) and presence (+S) of 100 µm AA (see **Figure** **3**A,B). mRNA expression analysis in Figure 3C demonstrates that the supplementation of 100 µm AA improved the expression of collagen type I (COL1A1) in human synovial on‐chip organoids over 14 days of maturation, independently of the hPL concentration, when compared to their respective non‐AA‐supplemented controls (1% hPL + S vs 1% hPL, p = 0.0853, 10% hPL + S vs 10% hPL, p = 0.3216). Notably, the presence of AA (+ S label in graphs) in 1% hPL further promoted a strong upward trend in collagen type III expressions (COL3A1), (1% hPL + S vs 1% hPL, p = 0.1412). This effect was less pronounced for AA supplemented 10% hPL, (10% hPL + S vs 10% hPL, p>0.9999). The presence of both COL1A1 and COL3A1 indicates the characteristic matrix constituents of reticular connective tissues, such as the synovium.^[^ ^29^, ^30^ ^]^ The mean expression of COL1A1 increased from 15 ± 11 (‐S) to 67 ±5 (+S) for 1% hPL, and 27 ± 12 (‐S) to 81 ± 43 (+S) for 10% hPL. COL3A1 expressions also increased with AA supplementation, rising from 59 ± 44 (‐S) to 224 ± 131 (+S) for 1% hPL, and 56 ± 40 (‐S) to 100 ± 21 (+S) for 10% hPL. However, the 1% hPL medium promoted a fourfold increase in reticular collagen type III expression, compared to a twofold increase for a 10% hPL‐containing medium. The 1% hPL medium formulation also induced a tendency toward higher lubricin expression (10 ± 17 (‐S) and 7 ± 10 (+S), p>0.9999 for 1% ± S vs 10% hPL ± S). CDH11 expression slightly increased from 17 ± 11 (‐S) to 19 ± 12 (+S), while VEGFA remained similar between treatments (2 ± 1 (‐S) to 1 ± 1 (+S)). COL3A1 expression for 10% hPL ± S showed little variation (1± 2 (‐S) vs 1 ± 1 (+S)), with p>0.9999 for all comparisons. CDH11 expression in 10% hPL decreased slightly (74 ± 98 (‐S) vs 1 ± 4 (+S), p>0.9999), while VEGFA expression also showed a slight decrease (5 ± 4 (‐S) vs 1 ± 1 (+S), p>0.9999 for all comparisons. Additionally, AA supplementation neither affected cell shape nor cellularity when generating the humanized synovial constructs under optimized maturation conditions, (Figure S4AB, Supporting Information). These results align with previous findings on the role of ascorbic acid^[^ ^31^, ^32^, ^33^, ^34^, ^35^ ^]^ that improved matrix gene expression levels in synovial models but did not modulate PRG4 expression, which is independent of AA metabolism. Notably, among tested conditions, the medium formulation containing AA with 1% hPL showed the lowest expression levels of pro‐inflammatory cytokines IL6 and CXCL8, as well as degradative proteases MMP1, MMP3, MMP13 and ADAMTS5 (Figure 3C lower panel). These catabolic changes underscore that the optimized non‐animal protocol for organoid maturation produced biopathologically inconspicuous organoids with a low‐level catabolic gene profile in our animal‐product‐free chip‐based synovia, particularly when used between weeks 3 and 6 after fresh tissue isolation (low passage number). Furthermore, the observed increase in catabolic OA marker gene expression with 10% hPL indicates that higher hPL concentrations may not necessarily yield improved outcomes in musculoskeletal bioengineering. This effect is likely due to the link between shifts in cell metabolism and pro‐inflammatory mechanisms, which are commonly observed in both degradative and fibroproliferative disorders. As a result, all subsequent microfluidic organoid cultures were generated using 1% hPL and 100 µm ascorbic acid in high‐glucose DMEM.

### 2.3 A Softer Fibrin Matrix Promotes Yap1 Overexpression that in Turn Indicates Pathological Mechanotransduction

**Figure 4:**
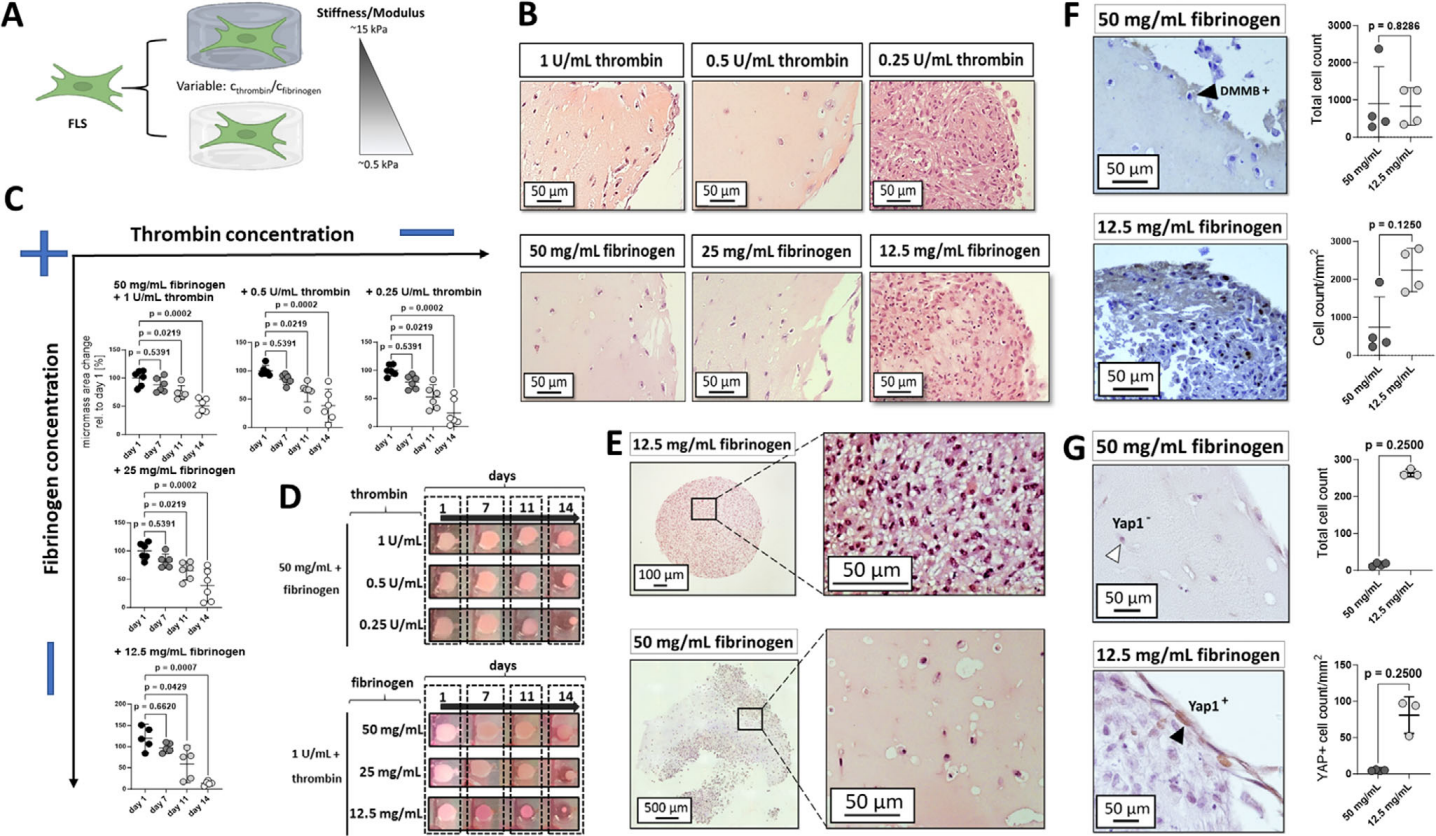
(A–D) Impact of TISSEEL matrix stiffness tuned by increase in thrombin concentration (0.25–1 U mL^−1^ thrombin with 50 mg mL^−1^ fibrinogen) and fibrinogen content (12.5 – 50 mg mL^−1^ fibrinogen with 1 U mL^−1^ thrombin) on (B) structural hallmarks of H&E‐stained non‐animal synovial biochip organoids at day 14 of maturation, as well as (C) condensation dynamics expressed as % area of day 1 for thrombin and fibrinogen concentration increase of the TISSEEL fibrin solution with (D) representative bright images. Data are expressed as mean with 95% CI with the Friedman test for multiple comparisons. (E,F) Brightfield images of (E) H&E and (F) DMMB (Methylene blue) stained non‐animal synovial organoids at day 14 post‐seeding with microscopic quantitation of the DMMB signal (n = 4). Data are expressed as % mean with 95% CI with unpaired Mann‐Whitney U analysis. (G) Microscopic analyses and quantitation of Yap1 immuno‐stained histological sections of day 14 non‐animal biochip organoids for the varying fibrinogen concentrations (n = 4) using a primary recombinant anti‐human Yap1 antibody (1:50). Data are expressed as % mean with 95% CI with unpaired Mann Whitney‐U analysis. The given sample number (n) always refers to biological replicates/different patient samples.

Since the structural integrity and the functionality of human synovial tissue are partially governed by its matrix proteins, the use of non‐human sources such as Matrigel can trigger non‐physiological cellular responses,^[^ ^36^, ^37^, ^38^ ^]^ leading to non‐tissue‐specific remodeling and altered biomechanical activities.^[^ ^39^ ^]^ In this work, TISSEEL fibrin hydrogel was studied as an alternative matrix to replicate a native synovial environment with enhanced tissue functionality, offering the ability to fine‐tune both physiological and pathological processes. Results in **Figure** **4**A,B show the response of organoid‐based primary synovial fibroblasts to TISSEEL fibrin hydrogels exhibiting an increasing dynamic modulus range of ≈0.5 – 30 kPa.^[^ ^40^ ^]^ Concurrently, the impact of both varying thrombin concentration and fibrinogen content on the condensation of synovial biochip organoids over 14 days using 1% hPL and 100 µm AA is illustrated in Figure 4B. The study results demonstrated that increasing the final thrombin concentration from 0.25 U to 1 U mL^−1^ in a hydrogel mix containing 50 mg mL^−1^ fibrinogen significantly and positively affected the relative area change of the organoids, reducing their volume variability within 14 days to ≈ 50%. In detail, organoid maturation stability decreased to ≈60% with 0.5 U mL^−1^ thrombin and further to 25% with 0.25 U mL^−1^ thrombin by day 14, see Figure 4C. When maintaining a thrombin concentration of 1 U mL^−1^ and reducing fibrinogen content from 50 to 12.5 mg mL^−1^, condensation dynamics of synovial organoids were enhanced over 14 days of maturation, leading to ≈39% for 25 mg mL^−1^ fibrinogen and 14% for 12.5 mg mL^−1^ fibrinogen, which still resulted in a clear separation into intima and subintima synovialis, particularly in the presence of 1 U mL^−1^ thrombin (see Figure 4B,E, lower image) resembling actual synovial tissue. Further reducing the fibrinogen content led to a loss of mechanical matrix stiffness, resulting in complete resorption of the fibrin matrix by the fibroblast‐like synoviocytes and the loss of synovial hallmarks, including the lining and sublining architecture (see Figure 4B,E, upper image). Surprisingly, these spheroid‐like constructs featured key characteristics of synovial hyperplasia or invasive pannus, including a high density of actively proliferating synoviocytes and giant cell‐like cells with round morphologies at the outermost layer. Histological image analysis of H&E‐stained paraffin‐embedded sections (Figure 4F) confirmed comparable cellularity (p = 0.8286) after two weeks of maturation in both the softer 12.5 mg mL^−1^ fibrinogen and denser 50 mg mL^−1^ fibrinogen matrices, each containing a final concentration of 1 U mL^−1^ thrombin. However, differences emerged in the lining‐specific cellularity between the two hydrogel variations, 50 and 12.5 mg mL^−1^ fibrinogen, both with 1 U mL^−1^ thrombin. A clear trend was observed, with an increasing cell count in the outer edge region of the organoids, ranging from ≈750 cells mm^−^^2^ for the 50 mg mL^−1^ hydrogel to ≈2250 cells mm^−^^2^ for the 12.5 mg mL^−1^ hydrogel (p = 0.2600). Another hallmark of a pro‐fibrotic environment, triggered by insufficient mechanical matrix stiffness and stability, is the upregulation of collagen synthesis, confirmed by an increase of Methylene blue^+^ stained cells, see Figure 4F. Next, the effect of matrix stiffness on Yap1 overexpression, a marker for pro‐inflammatory or pro‐fibrotic events in synovial tissue, was investigated through immuno‐histological analysis of whole‐organoid sections. Yap1 protein expression was nearly absent in organoids generated from 50 mg mL^−1^ fibrinogen (Figure 4G), while the more densely populated spheroid‐like models generated with the softer fibrin matrix exhibited a higher number of Yap1^+^ synoviocytes, particularly in regions of the outermost synovial lining. In summary, these matrix‐related effects suggest that a stiffer fibrin hydrogel can promote the physiological hallmarks of synovial tissue, while fibroblast‐like synoviocytes cultured in a softer (≈0.5 kPa modulus) fibrin matrix showed indications for a fibrotic synovial architecture.

### 2.4 Characterisation of Patient‐Derived Synovial Biochip Organoids over Four Cell Passages

**Figure 5:**
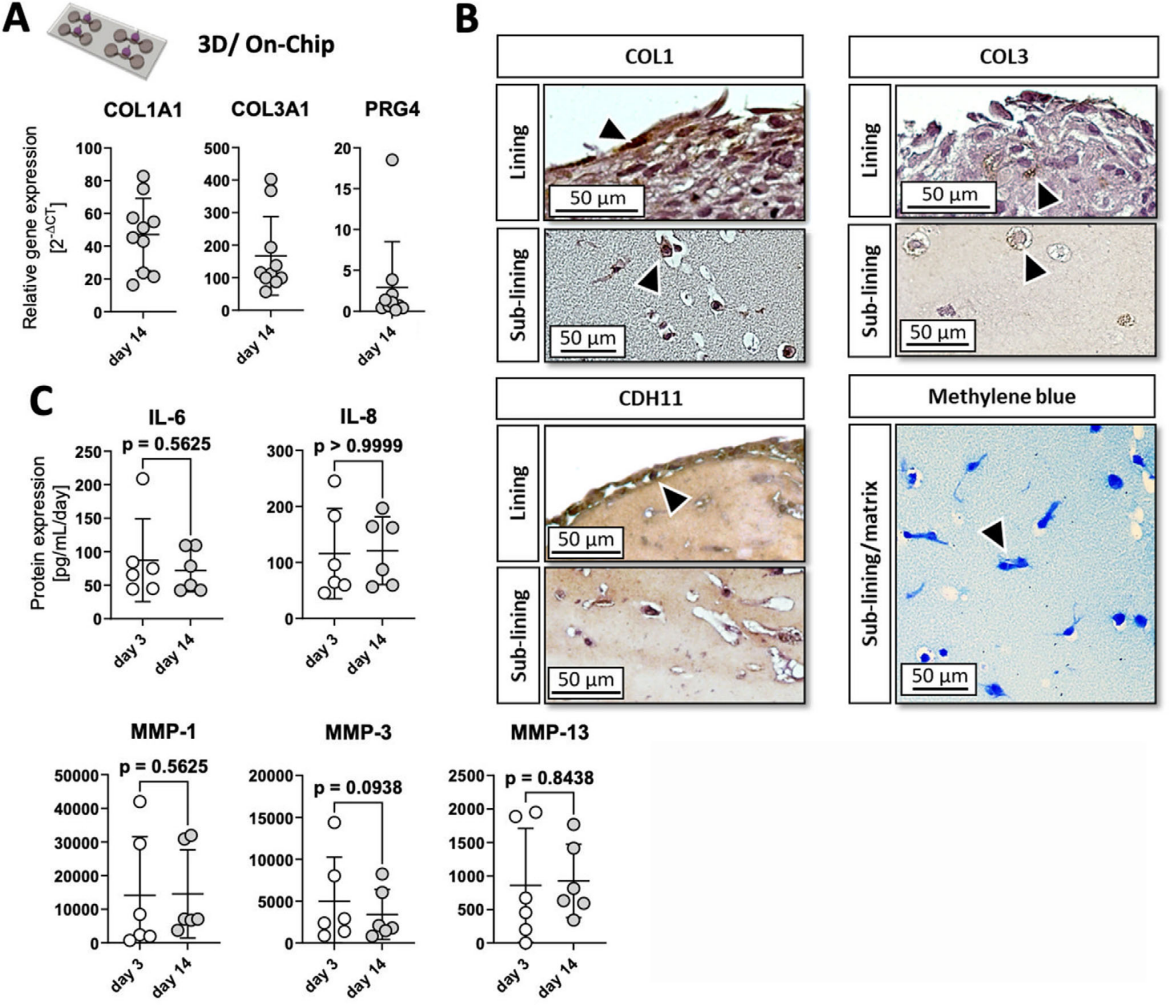
(A) Gene expression analysis of COL1A1, COL3A1, and PRG4 mRNA for primary OA patient‐derived non‐animal 3D biochip organoids after 14 days of maturation (n = 10, data are shown as mean with 95% CI) using the optimized culture conditions of 1% hPL and 0.1 mM ascorbic acid. (B) Histological evaluation of the regional location of collagen type I (COL1), collagen type III (COL3), cadherin‐11 (CDH11) as well as sulfated glycosaminoglycans (Methylene blue). Primary antibodies included recombinant anti‐human cadherin‐11 (1:50), collagen type I (1:50), and collagen type III (1:50). (C) Baseline secretion levels of OA hallmark proteins on days 3 and 14 of human OA synovial organoids during on‐chip culture (n = 6). Data are shown as mean with 95% CI with Wilcoxon matched‐pairs signed rank test. The given sample number (n) always refers to biological replicates/different patient samples.

Given the inherent patient variability of primary cell models, donor‐specific differences in matrix gene expression patterns were assessed in the next set of experiments. **Figure** **5**A shows patient‐dependent variability of selected physiological disease markers, including the expression levels of collagen type I, collagen type III, and lubricin (PRG4). Differences between individual patient‐derived organoids revealed a relative deviation of 0.44‐fold for COL1A1, 0.67‐fold for COL3A1 and 1.8‐fold (vs SDHA) for PRG4, demonstrating the importance of an OA patient‐centric approach in animal‐free disease modeling protocols. Additionally, the expression of collagen type I and III in both the lining and sublining regions were immuno‐histologically confirmed using 14‐day matured synovial constructs (see Figure 5B). Furthermore, the presence of intimal‐characteristic CDH11 was exclusively detected in the single‐cell layer forming the outer lining, while glycosaminoglycans were found evenly distributed throughout the matrix, as confirmed by Methylene blue staining. Further image analysis of the internal structure of OA‐FLS organoids revealed comparable cellularity throughout the constructs between day 7 and day 14 post‐seeding (Figure S5A,B, Supporting Information; p>0.9999) as well as between synovial organoids of six different biological FLS donors (Figure S5C, Supporting Information; p>0.9999). Further fluorescence image analysis showed neither significant variability between different z positions in synovial organoids (Figure S5D, Supporting Information; p = 0.700). Moreover, no significant difference in cellularity between commercial non‐OA and OA‐FLS origins could be observed (Figure S5E, Supporting Information; p = 0.8452).

Next, mRNA gene expression analysis was performed for organoids created over four passages of FLS from a single donor to investigate the effect of in vitro cell expansion on organoid marker expression. Figure S6A (Supporting Information) demonstrates a trend for expression reduction from passage at p4 (i.e., COL1A1, COL3A1, IL6, MMP3 and ADAMTS5 transcript levels), while a gradual decline was observable for PRG4 over increasing passages. Notably, changes over passages were also observable with FLS cultures of RA origins using long‐term cultivation protocols containing 10% fetal calf serum in previous studies.^[^ ^15^, ^41^, ^42^ ^]^ Targeted bulk RNA sequencing analysis (Figure S6B, Supporting Information) of our animal‐free synovial organoids of different passages and donors (n = 5) was analyzed against open‐access OA (label “OA”) and non‐OA synovial (label “Tissue”) tissue datasets. Relative higher z‐scores of NBN, CHEK1, MSH2, MSH6, and BRCA1/2 compared to non‐OA and OA synovial tissues indicate a higher stress response pattern than the transcriptomic in vivo stability, which is common for in vitro culture conditions where replicative stress can be attributed to DNA damage response gene upregulations and epigenetic shift.^[^ ^43^ ^]^ When comparing overall variabilities between organoids and the synovial tissues, the donor variability of organoids in between all the non‐OA and OA samples for a variety of transcriptomic stability and repair gene markers were within the observable z‐score ranges (e.g., variabilities in p53 or RAD51 or inflammation/fibrosis markers IL6, IL1B, ACTA2). Next, IPA™ pathway analysis of the five individual organoid data was conducted against the mean of the non‐OA tissue data to closely identify changes due to prolonged animal‐free cultivation and FLS passaging. Figure S6C (Supporting Information) confirms that patient variability is a stronger factor on transcriptomic stability pathways, and no passage‐dependent up‐ or downregulation of senescence, DNA damage, apoptosis or telomerase‐related pathways was observable with this analysis approach. Figure S7 (Supporting Information) shows additionally, that the gene expression analysis of fibroblast‐like synoviocytes (FLS) cultured as 2D monolayer revealed that OA‐ and non‐OA‐derived FLS at passage 3 exhibit similar transcriptional expression levels relating to extracellular matrix (COL1A1, COL3A1, and FN1), inflammation (IL6, CXCL8), matrix remodeling (MMP1, MMP3), and stromal identity (VCAM1, ACTA2), which demonstrates OA FLS utility to be used to form non‐inflamed 3D organoids under animal‐free conditions. Lastly, protein secretion analysis of a variety of disease‐relevant protein markers were investigated for a mixed population of n = 6 synovial organoids to elaborate whether the animal‐free culture conditions over a 14‐day period could affect the soluble “disease status” by not upregulating the secretion of degradative OA marker proteins (IL‐6, IL‐8, MMP‐1, MMP‐3, and MMP‐13, see Figure S5C, Supporting Information). The measured inflammatory cytokine release of IL‐6 and IL‐8 remained at an average level of 100 ± 20 pg mL^−1^ per day of culture (day 14 vs day 3, p = 0.5625, p>0.9999, respectively). Similarly, stable expression levels were observed for MMP‐1, ‐3, and ‐13, although at a higher magnitude (day 14 vs day 3, p = 0.5625, p = 0.0938, and p = 0.8438, respectively).

### 2.5 Stimulation of the Animal‐Product‐Free Synovium‐on‐a‐Chip Organoid with Picogram Levels of Cytokines Induces a Fibrotic Response Similar to Late‐Stage OA Tissue Hallmarks

**Figure 6:**
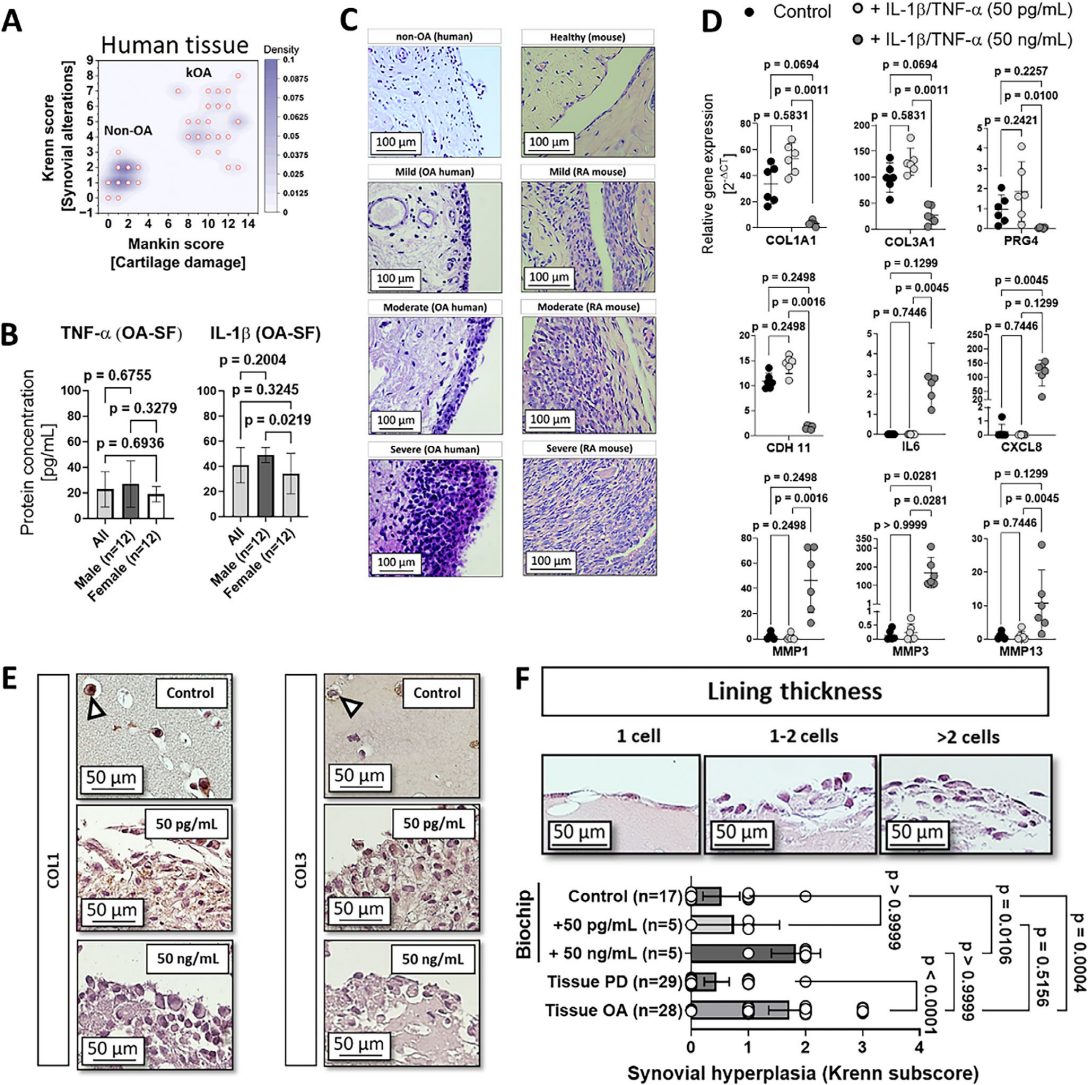
(A) Histological analysis of Krenn synovitis against Mankin cartilage degradation score for human non‐OA human synovial tissue from polydactyly (PD; n = 21) and kOA (n = 28) tissues. (B) Quantitation of IL‐1β and TNF‐α concentrations in synovial fluids of human OA patients (n = 24). (C) Representative images of human and murine synovial tissue sections of healthy (human cadaveric), mild to moderate human OA (TKA origin), as well as synovia from healthy and TNF‐α transgenic mice (1, 2 and 4 weeks). (D) Gene expression profile of non‐animal control organoids compared to OA organoids treated with 50 pg mL^−1^ or 50 ng mL^−1^ concentrations of IL‐1β and TNF‐α cytokine mix (n = 6) 14 days post‐maturation. (E) Representative brightfield images of collagen type I (COL1) and collagen type III (COL3) localization of control and treated OA non‐animal synovial organoids at day 14 post‐seeding. (F) Representative images of synovial organoid lining layer structures with lining layer thickening analysis (Synovial hyperplasia subscore of the Krenn scoring system) for non‐animal synovial organoids (n = 5‐17) in comparison to the synovial architecture of non‐OA human synovium from PD patients, PD (n = 28) and OA tissues (n = 29). Data are expressed as mean with 95% CI and depending on the normal distribution of single data points using the D'Agostino & Pearson test, data were analyzed upon significant differences between groups using either one‐way ANOVA and Tukey's post hoc for multiple comparisons (B) or the Kruskal‐Wallis test with Dunn's post hoc analysis for multiple comparisons. (D and F). The given sample number (n) always refers to biological replicates/different patient samples.

In OA research, nanogram‐level concentrations of pro‐inflammatory cytokines are often employed to trigger reproducible inflammatory pathways in vitro despite being considerably higher than physiological levels found in situ, as they are known to trigger severe or advanced stages of the disease. To follow this line of reasoning, in the final set of experiments, our animal‐product‐free microfluidic synovial organoids generated from a TISSEEL hydrogel mix containing 50 mg mL^−1^ fibrinogen and 1 U mL^−1^ thrombin in the presence of 1% hPL and 100 µm AA were challenged with low (chronic) and high (acute) levels of pro‐inflammatory cytokines to trigger OA‐relevant pathophenotypes for potential drug screening application. It is important to note that both synovial alterations (Krenn score) and cartilage damage (Mankin score) are elevated in OA patients (see **Figure** **6**A). In contrast, synovial alterations alone are prevalent in RA. Consequently, in this OA study, we investigated the protein levels of various pro‐inflammatory and pro‐fibrotic biomarkers from individual patients to gain deeper insights into the molecular microenvironment. Using a multiplex screening kit (n = 24), synovial fluids (SF) from OA patients, comprising a mixed‐sex sample population aged 50 to 84, were analyzed in separate experiments. As shown in Figure 6B, TNF‐α and IL‐1β, pro‐inflammatory cytokines commonly used as biochemical stimulants in OA in vitro models, were detected in the picogram range in kOA patient synovial fluid (SF) samples with 40.9 ± 14.0 pg mL^−1^ and 22.9 ± 13.7 pg mL^−1^, respectively. A more detailed overview of other fibrosis biomarkers, not relevant to the current investigation including pro‐inflammatory adipokines, IL‐6, or TIMP‐1, can be found in Figure S8 (Supporting Information). Based on the synovial fluid biomarker analysis, two distinct cytokine treatment groups were identified: a pathophysiological OA‐like group treated with a pg‐level cytokine cocktail (50 pg mL^−1^ of TNF‐α and IL‐1β, simulating chronic biomarker levels) and a supraphysiological group (simulating acute inflammatory events), which employed 50 ng mL^−1^ TNF‐α and IL‐1β. Furthermore, the presence of a mild, moderate, and chronic inflammatory milieu was also confirmed through histological investigations of the synovial structure and architecture in OA patient tissue, see Figure 6C.

Interestingly, synovial tissue biopsies of kOA patient origin showed progressive structural alterations with a gradual increase of the cell layer thickness of the intima synovialis and the absence of confluent immune cell infiltrates. These disease characteristics are absent in human, mouse, and equine non‐OA tissue samples exhibiting neither hypercellularity nor perivascular infiltration of myeloid and lymphoid cells (see also Figure S9A, Supporting Information). Results in Figure 6D demonstrate a pronounced pro‐inflammatory response in our animal‐product‐free microfluidic patient‐derived synovial organoids when stimulated with a 50 ng mL^−1^ cytokine cocktail. This included the upregulation of protease expression such as MMP1 (≈46‐fold, p = 0.2498 vs control), MMP3 (≈169‐fold, p = 0.0281), MMP13 (≈11‐fold, p = 0.1299), as well as a significant or strong upregulation of proinflammatory cytokines IL6 (threefold, p = 0.1299) and CXCL8 (≈115‐fold, p = 0.0045). Notably, compared to the untreated control, collagen expression levels including COL1A1 (≈30‐fold, p = 0.0694), COL3A1 (≈72‐fold, p = 0.0694), along with PRG4 (onefold, p = 0.2257) and CDH11 expression levels (ninefold, p = 0.2498) decreased in response to the strong inflammatory trigger, indicating the development of a pronounced inflammatory phenotype. Stimulation with a 50 pg mL^−1^ cytokine mix induced a significantly more moderate gene response in our microfluidic synovial organoids, closely resembling a mild OA in vivo condition. Specifically, gene expression levels of pro‐fibrotic COL1A1 were elevated (≈20‐fold; p = 0.5831), while COL3A expression similarly increased (≈31‐fold; p = 0.0694) in response to the treatment, compared to the untreated organoids. Pro‐inflammatory markers, including IL6 and CXCL8, and MMPs (MMP1, 3 and 13) were expressed at a comparatively low level, similar to those in the untreated control. Notably, the protease levels of the low trigger samples (50 pg mL^−1^) were many factors below the strong pro‐inflammatory cytokine treatment group (50 ng mL^−1^), indicating low protease gene expression levels closer to untreated than inflamed organoids. Moreover, CDH11 was slightly overexpressed (threefold; p = 0.2498) when cells were exposed to low pro‐inflammatory stimuli. The expression of the intima synovialis characteristic lubricin (PRG4) remained comparable to the expression level of the control (onefold increase, p = 0.2421) when challenged with the low‐level pro‐inflammatory trigger. These findings suggest that the animal‐product‐free microfluidic patient‐derived organoids can develop typical human characteristics of OA synovial tissues when cells were stimulated with cytokines on‐chip.

To confirm these characteristic changes at the structural level, the divergent matrix effects resulting from pro‐inflammatory cytokine treatments were also verified at the protein level, as demonstrated in Figure 6E for both collagen type I and type III staining. Additionally, the basic Krenn scoring system for grading lining layer thickness was used to evaluate and compare synovitis in late‐stage fibrotic and severely inflamed OA synovial organoids. Results shown in Figure 6F confirm synovial hyperplasia, synovial thickening, and corresponding gene expression and matrix divergencies. Here, the synovial lining layer hyperplasia and outermost layer thickening were directly proportional (control < pg mL^−1^ < ng mL^−1^) to the amount of administered pro‐inflammatory cytokine. The higher, ng‐level biochemical stimulus activated synovial‐fibroblast within the organoid construct more strongly (p = 0.0106 vs control) compared to the lower pg‐level trigger (p>0.9999 vs control). The strong resemblance of lining cellularity between the strong trigger‐induced model and the actual OA patient tissue (ng/mL vs OA tissue, p>0.9999) underscores the lining thickness as a valuable and translatable tissue biomarker, effectively bridging in vivo tissue pathology with in vitro tissue architecture.

**Figure 7:**
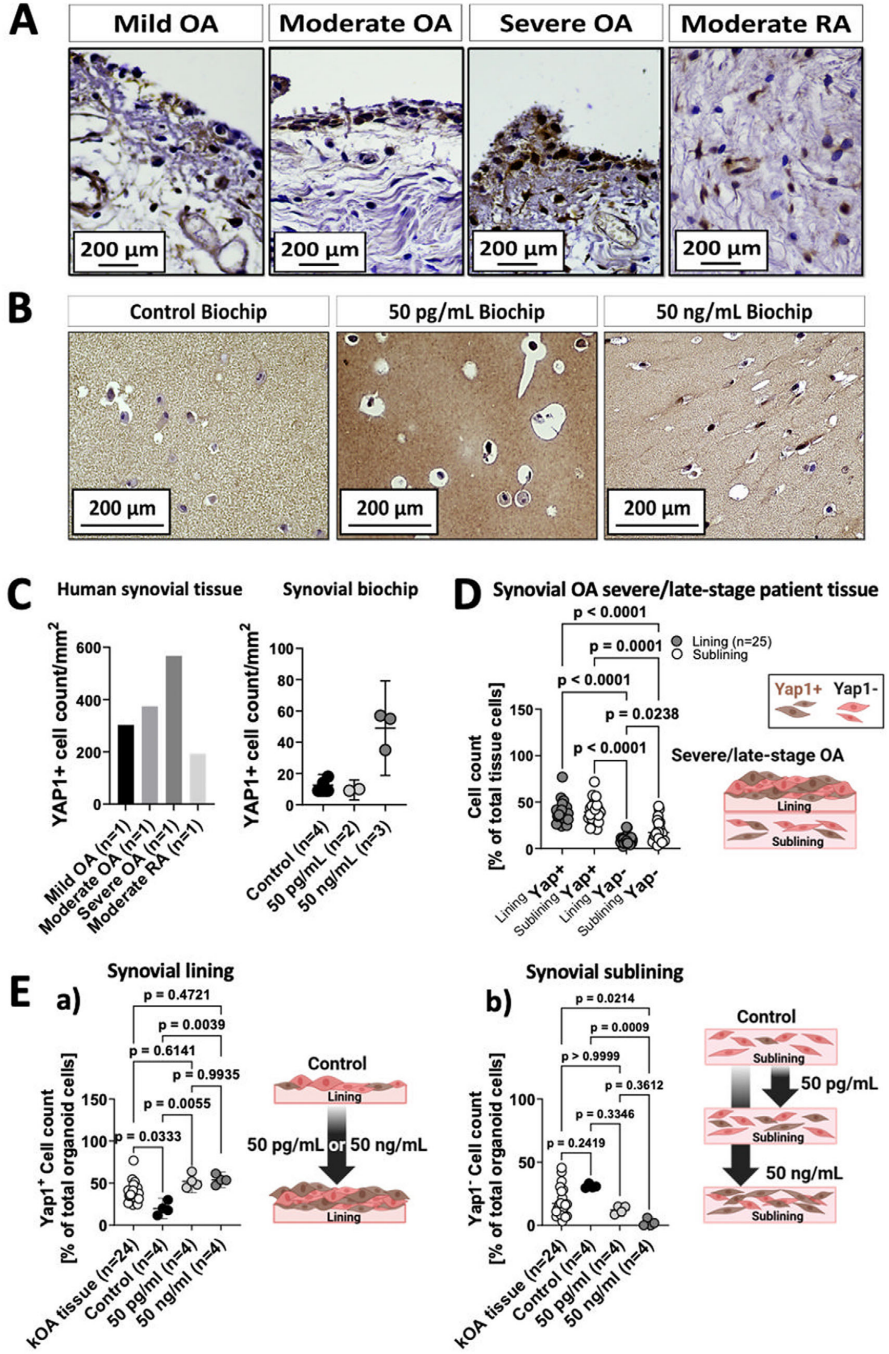
(A) Representative images of Yap1^+^ synoviocytes in the lining and sublining regions of human mild to severe OA and moderate RA tissue specimens. (B) Representative images of Yap1^+^ synoviocytes in the sublining of healthy non‐animal synovial biochip organoids in comparison to OA organoids treated with either 50 pg or ng/mL IL‐1β and TNF‐α solution at day 14 post‐maturation. (C) Quantitative analysis of Yap1^+^ cell distribution in the context of increasing disease severity in kOA synovial patient tissue (n = 1) and synovial biochips (n = 4, 2 or 3). (D) Image‐based quantitation of Yap1‐stained synoviocytes of immuno‐stained histological lining and sublining regions of OA‐patient derived synovial tissues (n = 24) and a schematic drawing of lining‐ and sublining‐specific distribution of Yap1‐positive and negative cells in late‐stage synovial OA patient tissue. (E) Microscopic analyses and quantitation of Yap1 in the lining (a) and sublining (b) regions of day 14 non‐animal biochip organoids, including control, as well as OA organoid specimens treated with 50 pg or ng/mL IL‐1β and TNF‐α cocktail (n = 4). Data are expressed as mean with 95% CI and were tested for normal distribution using the D'Agostino & Pearson test and significant differences were tested either with one‐way ANOVA (D) with mixed effect analysis and Geisser‐Greenhouse correction with Tukey's multiple comparisons tests, a two‐way ANOVA with a Tukey's post hoc analysis (E a and b). The given sample number (n) always refers to biological replicates/different patient samples.

In the final set of experiments, Yap1 expression was again investigated to establish its correlation with the severity of late‐stage OA synovial tissue alterations. **Figure** **7**A demonstrates that Yap1 expression in OA tissues was predominantly localized at the intima synovialis and progressively increased with the severity of hyperplasia. Additionally, structural tissue analysis of human RA revealed a confluent localization of Yap1+ cells in fibrous sub‐intimal regions. Both findings indicate that Yap1 is expressed by synoviocytes originating from both OA and RA, irrespective of the specific regions affected by these diseases. Furthermore, an increase in Yap1+ cells is correlated with disease severity. Yap1 localization was also studied for untreated control and inflamed synovial organoids, as shown in Figure 7B. Control organoids exhibited only a sparse number of Yap1^+^ cells, whereas organoids treated with pg‐level cytokines showed that almost all cells were stained positive for Yap1, see Figure S9B (Supporting Information) for additional staining controls. A similar effect was observed for ng‐level of cytokines, almost all cells, independent of intimal or sub‐intimal location showed a strong Yap1 positive staining. The quantitative analysis in Figure 7C, comparing Yap1^+^ cells in histological OA synovium samples (see also Figure 7A) with increasing disease severity, consistently matched the increasing count of Yap1 expressing cells in synovial biochips (Figure 7B), pointing out the potential of this in vivo marker as an in vitro translatable disease measure in the human synovial biochip.

Lastly, the spatial distribution of Yap1‐positive cells within the lining and sublining regions of synovial OA patient tissue as well as organoids was investigated. This analysis was intended to generate a landscape of Yap1‐expressing cells and to serve as an in vivo OA control to be compared with the prevailing conditions in the final biochip model. In synovial tissue of kOA patients, an equal distribution of Yap1^+^ cells was evident throughout the lining (38.4 ± 11.4%) and sublining (39.4 ± 11.9%) regions of the total cell count, see Figure 7D. In detail, the lining layers showed a lower number of Yap1 negative cells (9.1 ± 4.9%) in contrast to the sublining (17.3 ± 11.6%) of the total cell count (p = 0.0231). This finding suggests a widespread presence of Yap^+^ cells across both the lining and sublining areas in late‐stage OA, with a notable increase in cellularity, particularly in the sublining region. The observable and progressive increase in YAP⁺ cells observed in OA patient tissues (Figure 7C), together with the region‐independent distribution of YAP expressing synoviocytes (Figure 7D), suggests that the elevated YAP⁺ cell counts in osteoarthritic synovium may primarily reflect enhanced pathological mechanisms of cell activation and mechano‐signalling in both the lining and sublining regions.

To generate a human synovial model showing the highest degree of comparability on a tissue‐like level, it was essential in the current study to compare Yap positivity in the lining and sublining cells of the synovial biochip model with those observed in actual OA synovial tissue samples. The comparative analysis of synovial biochip organoids in Figure 7E (a) points out a higher amount of YAP1^+^ cells in the lining layer with +18.6% for kOA tissue, +32.4% for 50 pg mL^−1^, and +34.2% for 50 ng mL^−1^ compared to the untreated control biochips (p = 0.0333 for kOA tissue vs control, p = 0.0055 for 50 pg mL^−1^ vs control and p = 0.0039 for 50 ng mL^−1^ vs control). Conversely, a strong comparability was observed between kOA tissue and the two treatment groups (p = 0.6353 and p = 0.3921 for pg mL^−1^ and ng mL^−1^ vs kOA tissue, respectively). Notably, both treatment modalities showed a similar distribution of Yap1^+^ cells in the lining layers of synovial organoids (p = 0.9935). A similar increase of Yap1^+^cell populations was observed in the sublining regions of the biochip organoids, with a slightly higher increase in response to the stronger treatment of 50 ng mL^−1^. This resembled Yap1^+^ sublining cellularity more closely (39.4% ± 121 for kOA tissue, 10.1% ± 5.2 for the biochip control, 35.1% ± 5.4 for 50 pg mL^−1^, and 45.4% ± 6.1 for 50 ng mL^−1^), (Figure S10A, Supporting Information). When comparing the distributions of Yap1^−^ cell populations of synovial tissue and synovial organoids (Figure 7E (b)) within the sublining region, the 50 pg mL^−1^ treatment group showed better agreement with actual tissue (12.2% ± 6.1 for 50 pg mL^−1^ and 17.3% ± 12.0 for kOA tissue), while the organoids treated with the 50 ng mL^−1^ treatment regime showed a depletion of Yap1 negative cells dropping to ≈1.9 ± 2.8%. For Yap1^−^ cells in the lining (Figure S10B, Supporting Information), both cytokine trigger groups (50 pg mL^−1^ and 50 ng mL^−1^) and kOA tissue displayed a low abundance of this cell population, with ≈9.1% ± 5.1 for kOA tissue, 1% ± 2.0 for 50 pg mL^−1^, and 1.9% ± 2.0 for 50 ng mL^−1^. These findings for the cellular distribution of Yap^+^ cells in the lining and sublining regions of the untreated biochip synovium established a baseline, serving as the “healthy / non‐OA control” for future experiments. Results also demonstrate that Yap1 is increasingly expressed both, in naturally developed in vivo conditions and in cytokine‐triggered in vitro disease models. Moreover, the overall distribution of Yap1^+^ and Yap1^−^ cells in the lining and sublining regions of kOA synovial patient tissue was successfully replicated in the human synovial biochip model.

## 3 Conclusion

The replacement of commonly used animal‐derived products like Matrigel and FCS with suitable human‐derived or synthetic alternatives in advanced in vitro cell culture is essential for creating clinically relevant human disease models with clean science approaches. In osteoarthritis, (patho)physiological cell‐to‐cell and cell‐to‐matrix interactions are largely governed by circulatory factors, serum proteins, and matrix composition, all of which influence disease onset, progression, and remission. Thus, using animal‐derived products in human cell culture protocols remains problematic and suboptimal, as it questions the authenticity and validity of the resulting tissue or disease models. Before animal‐cell and animal‐product‐free in vitro culture methods can be widely used to establish human disease models for OA research, reliable and standardized protocols must first be developed. The primary goal of this study was to create a fully human synovial organoid that mimics the key characteristics of healthy synovial architecture. Results demonstrated that with low human platelet (hPL) concentrations (1%) and ascorbic acid, patient‐derived synovial organoids generated from low passage fibroblast cultures displayed a physiological phenotype closely resembling human synovial architecture. This included an outer intima synovialis facing the synovial fluid and a loose subintimal network of connective tissue. This is an important detail of our study to highlight the usage of lower passage cells isolated from primary synovia, because previous genetic and karyotype studies on primary fibroblasts already indicated a decline of genetic stability and chromosomal aberrations after passage 7 to 8.^[^ ^44^, ^45^, ^46^ ^]^ Interestingly, a tenfold increase in hPL (10%) activated synovial fibroblasts, as evidenced by elevated markers of an arthritic phenotype, including IL6, CXCL8, CDH11, MMPs, and ADAMTS5. Under physiological mechanobiological stress (≈30 kPa Young's modulus) with stiff fibrin scaffolds (50 mg mL^−1^), near‐normal organoid responses were observed. In contrast, softening the matrix (≈0.5–1 kPa, similar to Matrigel) induced Yap1 expression in synovial fibroblasts in the synovial lining, a strong indicator of pathological response. This finding is consistent with Yap1 expression profiles in patients with diabetic osteoarthritis and rheumatoid arthritis.^[^ ^47^, ^48^, ^49^, ^50^, ^51^, ^52^, ^53^ ^]^ Notably, our animal‐product‐free synovial tissue construct closely matched human tissue in collagen type I, reticular collagen type III, and lubricin matrix expression. Thus, fully functional 3D synovial membranes with physiological lining and sublining layers can be successfully generated using our optimized animal‐product‐free 3D culture protocol.

To assess the capability of our animal‐product‐free synovium‐on‐a‐chip organoid culture as a patient‐specific microfluidic OA disease model, we challenged the biochip organoids with proinflammatory cytokines, including IL‐1β and TNF‐α. Treatment with in vivo cytokine levels (e.g., picogram range) from knee OA patient synovial fluid induced a pro‐fibrotic pattern, including upregulation of COL1A1, COL3A1, and CDH11. In contrast, exposure to a strong inflammatory environment (e.g., microgram range) led to a significant inflammatory response, marked by elevated IL6, CXCL8, and MMPs. Additionally, exposure to low cytokine concentrations also promoted synovial extracellular matrix (ECM) production and strong remodeling, without inducing large‐scale inflammatory responses. Here, the upregulation of cadherin‐11, a key mediator of synovial migration and invasiveness, aligned with previous findings related to the synovial RA pathophenotype.^[^ ^54^, ^55^, ^56^ ^]^ Moreover, a direct comparison between our in vitro synovial organoids and osteoarthritic patient synovia, which exhibited strong responses at the synovial intima, revealed a strong correlation in terms of architectural and structural variability, while the cellularity of the subintima was not affected. In contrast, rheumatoid synovia of a previously reported transgenic rheumatic arthritis model displayed confluent hypercellularity, similar to the response observed in our organoids using the softer TISSEEL hydrogel compositions. Our findings suggest that synovial hyperplasia is a hallmark of late‐stage knee OA,^[^ ^28^ ^]^ a feature traditionally linked to RA. Thus, sub‐intimal confluency and hypercellularity can serve as distinguishing architectural and structural features between RA and OA patient tissues. Comparing current results from our OA model with previously published RA micromasses,^[^ ^14^, ^54^, ^55^ ^]^ we concluded that sub‐intimal structural changes in the synovial network are a more reliable indicator of disease progression than in the case of OA patient tissues. Importantly, we observed an increase in Yap1^+^ cells during low‐level pro‐inflammatory cytokine treatment, a marker typically associated with RA pathology rather than OA. Our results further show that the presence of Yap1^+^ cells is not exclusive to high‐level inflammation in RA,^[^ ^52^ ^]^ but is also observed in the intima synovialis of knee OA specimens. This is important since Yap1 is also considered a pathological marker in various inflammatory diseases, including atherosclerosis (e.g., Yap1 suppression is connected to NLRP3 inflammasome inhibition^[^ ^48^ ^]^) and osseotendinous inflammation (e.g., Yap/Hippo pathway is linked to excessive tenascin^[^ ^51^ ^]^). Notably, we could demonstrate that, unlike Yap1 activity in RA FLS,^[^ ^50^ ^]^ the response of osteoarthritic FLS isolated from patient‐derived synovial tissue better resembles OA synovia when stimulated with low concentrations of pro‐inflammatory cytokines. While the mechanistic role of Yap1 in regulating cartilage dysfunction has been explored,^[^ ^57^ ^]^ our osteoarthritic synovial organoid study could confirm a poorly understood role of Yap1 in osteoarthritis synovia. Modulation of Yap1's transcriptional influence can constitute a strategy to attenuate degradative and inflammatory processes in the synovium‐cartilage axis.

The limitation of the current study is the utilization of primary cells, which are prone to inter‐sample and donor variabilities, which renders this technology less optimal for the generation of highly reproducible synovial models that aim for screening applications. Regarding the animal‐free culture conditions, the observed transcriptional patterns in organoids that included upregulation of DNA damage response (DDR) and genomic stability markers alongside suppressed inflammatory gene expression reflect an adaptive response pattern to the current 3D culture conditions. While this combination of 1% hPL, 1% ITS, and 100 µM ascorbic acid supports calf serum‐free culture and promotes essential anabolic and antioxidative pathways, our data suggest that under the current optimized conditions, organoids experience low levels of proliferative/oxidative stress. This could potentially lead to a phenotypic drift, reducing the potential of the organoid culture protocols for long‐term biological usability. While the low expression of inflammatory markers suggests a beneficial suppression of unwanted inflammation, the consistent DDR activation highlights the requirement to better control culture‐induced stress responses in future optimizations. Evaluation of alternative xeno‐free and/or synthetic media replacements as well as investigation of additional antioxidant supplementations in combination with mechanistic stress marker monitoring for synovial fibroblasts will improve our understanding of passaging effects on primary 3D synovial organoids in the context of tissue‐mimetic functionality. In the same line of thought, future improvements on stem‐cell and iPSC‐derived models of synovial tissue^[^ ^19^, ^58^, ^59^ ^]^ may bridge this gap by providing stabilized and more standardized synovial models, which can be easily adopted to our tissue‐mimetic organoid biochip generation protocols established in the current study. Notably, such cells can better provide healthy‐origin synoviocytes, which potentially address the challenge of scarcity of non‐OA healthy synovial tissue biopsies and cells in joint‐on‐a‐chip studies that focus on OA onset and progression.

In conclusion, our study demonstrates that primary mature stromal cells such as fibroblast‐like synoviocytes (FLS) derived from easily accessible late‐stage OA patient tissue, can achieve a non‐inflamed organoid status using commercially available non‐animal culture and maturation products for in vitro generation of organoids without using steroids.^[^ ^55^, ^60^, ^61^ ^]^ To our knowledge, this is the first study to establish an entirely human, tissue‐mimetic osteoarthritic synovial biochip model devoid of any animal‐derived products, exhibiting molecular and cellular secretion patterns comparable to those found in OA patients when exposed to physiological levels of cytokines. We strongly believe that our animal‐product‐free, patient‐centric approach more accurately resembles the synovial architecture and structural mechanisms observed in OA. In particular, the role of structure‐function relationships (i.e., cartilage or synovial organoids) has been largely overlooked in earlier studies investigating the molecular processes of OA in vitro, using animal‐derived or animal‐product‐derived 3D tissue models.^[^ ^62^, ^63^, ^64^, ^65^, ^66^, ^67^, ^68^ ^]^ Overall, this study highlights the potential of fully human, animal‐product‐free organoid systems as a promising tool for a better understanding of the underlying molecular and mechanical pathways using joint‐on‐a‐chip.^[^ ^8^, ^69^, ^70^, ^71^, ^72^ ^]^ This in turn will allow, in our opinion, improved modelling of different pathotypes and, in the long run, the development of stratified preclinical synovial joint OA models as disruptive technologies for not only basic research but also improved drug screening studies.^[^ ^15^, ^59^, ^73^, ^74^, ^75^, ^76^, ^77^, ^78^ ^]^

## 4 Experimental Section

### Isolation of Primary kOA Synoviocytes and Cell Culture

Primary fibroblast‐like synoviocytes (FLS) were isolated from kOA patient‐derived synovial specimens collected during total knee endoprosthesis surgeries. Patients were preselected based on OA severity, with Kellgren‐Lawrence scores of 3 to 4, and an age range of 55 to 80 years from a mixed‐gender pool. Ethical approval was granted by the Medical University of Vienna (1822/2017 and 1034/2020), and informed consent was obtained from all participants. Synovial tissue fragments were cultured in Dulbecco's modified Eagle Medium (DMEM; high glucose, Gibco) supplemented with 10% fibrinogen‐depleted human platelet lysate (hPL; Elarem FD, PL Bioscience) and

1% antibacterial and antifungal agent Anti/Anti (Gibco). Cultures were maintained at 37 °C in a humidified atmosphere with 5% CO₂, and cells were subcultured at 70–80% confluency using TrypLE Express enzyme (Thermo Fisher), a recombinant and animal‐free dissociation reagent. Fresh synovial monolayer cultures were split in a 1:2 ratio weekly and subcultured until further use. For comparative analyses commercially available, normal human synovial fibroblasts (n = 3 donors), isolated from non‐OA synovial tissue, were purchased from INNOPROT.

### Biochip Fabrication and On‐Chip Culture

Microfluidic biochips were fabricated using xurographic prototyping with a CAMM‐1 plotter (Roland) as previously described.^[^ ^15^, ^79^ ^]^ In brief, plasma bonding (Harrick Plasma) was applied to adhere chip layers, followed by annealing at 80 °C overnight to ensure tight bonding. The chip surface was treated with 0.5 wt% ethanolic LIPIDURE CM5206 solution to prevent cell adhesion and promote organoid formation. UV treatment was applied 24 h before organoid culture for device sterilization.

Synovial organoids were prepared by mixing 30 µL of reconstituted TISSEEL (Baxter) fibrinogen solution (100 mg mL^−1^) with 15 µL of FLS suspension (1.2 × 10⁷ cells mL^−1^) in hPL‐free DMEM (1:4 mixing ratio). The mixture was combined with 15 µL thrombin solution (1–4 U mL^−1^), and 45 µL of the resulting fibrin hydrogel was injected into individual biochip units. After polymerization for 45 min at 37 °C, hydrogel ports were sealed with PCR adhesive foil strips. The culture medium, supplemented with 1% ITS (Gibco) and 1% NEAAs (Gibco), was added to each biochip. Medium changes were performed on days 3, 7, and 10, with culture endpoints on day 14, (a graphical scheme of the optimized protocol for generation, maturation and analysis pipeline can be seen in Figure S1, Supporting Information). Variations in hPL concentrations (1–10%) and the addition of 100 µM ascorbic acid (Sigma Aldrich) were employed for organoid optimization. Pro‐inflammatory conditions were simulated by adding 50 pg mL^−1^ or 50 ng mL^−1^ human recombinant TNF‐α and IL‐1β (Biolegend) to the culture medium over two weeks, with untreated samples serving as controls.

### Metabolic Activity Assay

Metabolic activity was assessed using the PrestoBlue assay (Thermo Fisher). Standard curves for hPL concentrations (10%, 1%, and 0.1%) were generated using 20 × 10^3^ cells from 11 individual patients at a serial dilution of 1:2. For a time course of seven days the esterase activity was monitored by medium replacement with hPL‐free DMEM with 10% assay reagent. After 30 min incubation fluorescence intensity was captured using a Perkin Elmer plate reader (Em 550/Ex 610 nm) for both 2D monolayer cultures and 3D micromasses in 48‐well plates.

### RT‐qPCR Gene Expression Analysis

After washing the samples with 1× PBS, mRNA isolation from 2D and 3D cultures and cDNA synthesis were performed. In brief, 350 µl RLT lysis buffer (IST Innuscreen), which was additionally supplemented with v/v 1% 2‐Mercaptoethanol (β‐ME, Sigma Aldrich) for organoid lysis, was used to prepare 30 µL aqueous mRNA solution according to the manufacturers’ instructions using the innuPREP RNA Mini Kit. The NanoDrop 2000 system was applied to quantify purity and the amount of mRNA. The cDNA synthesis was performed using the High‐Capacity Reverse Transcription Kit (Applied Biosystems). The quantitative real‐time PCR was conducted using a qPCR system (QuantStudio 3, Applied Biosystems) and a SYBR Green Master Mix. Specific primers for the target genes, (see **Table** **1**) were self‐designed using Primer‐BLAST freeware (NCBI). The qPCR protocol followed the manufacturer's instructions (Power Track SYBR Green Master Mix, A46109, Applied Biosystems). Amplification cycles were set to 40 rounds, followed by a melt curve analysis to confirm the specificity of the amplification. For gene expression analysis, Ct values were normalized relative to the expression of a housekeeping gene (SDHA), and consecutively calculated 2^^−ΔCt^ values were used to determine basic target‐specific expression levels and differences in expression among treatment groups including untreated control. All qPCR experiments were conducted in duplicate technical replications.

**Table 1:** Used primers for RT‐qPCR‐supported gene expression analysis.

Gene	Forward primer [5′ – 3′]	Reverse primer [3′ – 5′]
SDHA	TGGGAACAAGAGGGCATCTG	CCACCACTGCATCAAATTCATG
COL1A1	CACTGGTGATGCTGGTCCTG	CGAGGTCACGGTCACGAAC
COL3A1	GAAAGAGGATCTGAGGGCTCC	AAACCGCCAGCTTTTTCACC
PRG4	ATGGAGTGCTGCCCTGATTT	GATGTGGGATTATGCACTTCTGC
IL6	ATAGGACTGGAGATGTCTGAGG	AGGCAACTGGACCGAAGG
CXCL8	CAAACTTTCAGAGACAGCAGAG	CAGTGAGATGGTTCCTTCCG
CDH11	TCAGATGGGTGGAGTGTGTT	ACGCAGACCTCTCTTGGGAT
VEGFA	CCTTGCCTTGCTGCTCTA	TTCTGCCCTCCTCCTTCT
MMP1	TCTAGAAACACAAGAGCAAGATGTG	GCGTGTAATTTTCAATCCTGTAGGT
MMP3	AGTCCCTCTATGGACCTCCC	AGGGATTTGCGCCAAAAGTG
MMP13	TGAGCTGGACTCATTGTCGG	GAACCCCGCATCTTGGCTT
ADAMTS5	GCTGCAGTATGACAAGTGCG	TCACCACGTCAGTGTAACCC
HAS1	GGCCTGGTACAACCAGAAGT	GACCTGGAGTGTACTTGGTAG
VCAM1	GGAAATGACCTTCATCCCTACCA	CTCTGGGGGCAACATTGACA
CD55	CAAATGCTCAAGCAACACGGA	TGAAACACGTGTGCCCAGAT
ACTA2	CCCCGGGAGCCAGTCT	GCTTCACAGGATTCCCGTCT
FN1	ACGCGCTTAAAGGACCCAAT	ATCCATCCCCACAGGAATGC
RUNX2	CTTGTGGCTGTTGTGATGC	CTGTTGCTGCTGCTGTTG

### Luminex Assay for Protein Quantification

Cell culture supernatants were collected and stored at −80 °C until further processing. The samples were analyzed for the secretion of proteins, including IL‐6, IL‐8, MMP‐1, MMP‐3, MMP‐13 (panel 1) and TIMP‐1, Adiponectin, TNF‐α and IL‐1β (panel 2). Before performing the Luminex assay, the samples were brought to room temperature and diluted if necessary to achieve the optimal measurement range for the assay. The Luminex assay was conducted using a fluorescence‐based multiplex bead system. The assay was performed according to the manufacturer's instructions (R&D Systems) and their recommendations. Samples were centrifuged, diluted, and then incubated with the beads on a shaker at room temperature for 1–2 h, followed by washing steps. For signal development, incubation with a biotin‐antibody and streptavidin‐PE cocktail was repeated. Target binding was subsequently measured using a MAGPIX system, and the results were expressed in µg/mL. For measurement and data analysis, standardized calibration curves were used to determine the concentration of target proteins in the samples. The data were analyzed using the appropriate analysis software (e.g., xPONENT Software), and the results were presented as mean ± standard deviation across technical replicates.

### Histology and Microscopic Analysis

After medium removal, the cell‐laden hydrogel constructs were fixated by adding ≈150 µL of 7.5% formaldehyde (1 L, neutral, buffered, SAV Liquid Production GmbH). After pre‐fixation for ≈1 h on‐chip to support structural stability of the constructs, cell‐containing hydrogel clots were removed with disposable tweezers from each organoid compartment after removal of the top lid with a scalpel, and immersed in 500 µL of 7.5% formaldehyde for at least another 24 h at room temperature (RT). The clots were treated with increasing concentrations of denatured alcohol (70%, 80%, 90%, and 100%) and incubated at each step for 1–2 h. Dehydrated clots were incubated for 1 h in an intermediate medium (1L Xylol, Merck and immersed in paraffin (Paraplast Plus, Leica). Embedded clots were cut in a thickness of 2.5 µm using a microtome (Thermo Scientific Finesse HE+). The obtained specimens were pulled on microscope slides, dried over night at 60 °C and stained with hematoxylin (500 mL, Papanicolaou's solution 1a Harris’ hematoxylin solution; Merck Millipore) and eosin (100 mL dH_2_0, 400 mL 96% alcohol, 5 g eosin G (Certistain, Merck Millipore) by applying the following procedure: a) de‐paraffinization with 2x immersion for 10 min in Xylol, 100%, 96%, 80%, and 70% alcohol denat., and dH_2_0, b) incubation in hematoxylin solution for 10 min, c) flushing with tap water for 5 min, d) immersion in 0.1% hydrochloric acid alcohol for 1 min, e) flushing with tap water for 1 dip, f) immersion in aqueous ammonia solution for ≈ 10 dips, g) immersion in 70% alcohol for 1–5 min, h) incubation in eosin solution for 1–2 min, i) washing with increasing concentrations of alcohol of 70%, 96%, and 100%, for 1–5 min each, j) immersion in xylol for 5 min and k) mounting of the H&E stained specimen with Eukitt (500 mL, Eukitt mounting medium, Orsalic GmbH).

For Mankin scoring, 2.5 µm thick paraffin sections of tissues were deparaffinized and stained 24 h with ethanolic Safranin O (2 g per 300 mL of 33% ethanol solution; Sigma‐Aldrich), multiple 1.2% picric acid dippings (VWR) and counterstaining for 30 s using 0.2% light green Goldner III solution (Morphisto, Frankfurt, Germany) with dH_2_0 rinsing steps in between.

For dimethylmethylene blue (DMMB) staining, deparaffinized sections were incubated with 1% DMMB (Sigma Aldrich) for 15 min, followed by a rinse in distilled water for 5 min.

After dipping the sections twice in 70% alcohol for 5 seconds, once in 96% alcohol for 5 seconds and isopropanol (2‐propanol) for 10 seconds, the sections were treated with xylene for 10 min before mounting with Eukitt.

For immunostaining of sections, deparaffinized sections were antigen recovered in citrate buffer (2.101 g citric acid per liter dH_2_0, pH 6; Merck) for 10 min in the microwave (150 watts) for Yap1 and Cadherin 11, or Proteinase K (Merck) for 15 min at 37 °C for collagen type I and III and washed after initial buffer use 2x in 1 × PBS before incubation for 3 min in hydrogen peroxide (H_2_O_2_) solution (3% v/v, Merck) and repeated washing twice with 1 × PBS. Before using primary antibodies were prepared in 10% goat serum for anti‐human Yap1 (1:50, recombinant anti‐rabbit, Abcam, ab205270) and in 10% horse serum for CDH11 (1:200, anti‐mouse, R & D Systems, MAB 1790), collagen type I (1:1000, anti‐goat, Southern Biotech, 1310‐01) and collagen type III (1:250, anti‐mouse, Abcam, ab6310) were incubated overnight in a staining chamber at 4 °C. A biotinylated secondary antibody (biotinylated IgG, Vector) was incubated for 30 min at room temperature, washed twice in 1× PBS, and incubated with ABC‐Elite (Vectastain ABC kit, Vector) for another 30 min. The DAB colour reaction was produced by incubation with 3′3‐diaminobenzidine tetrahydrochloride hydrate (0.25 g in 250 mL dH_2_0 and 15 µL 30% H_2_O_2_, Fluka) for 6 min before washing in dH_2_0 and nuclei counterstaining with Mayer's hemalum (Merck).

### Statistical Method Section

Statistical analysis was performed using Prism 9 and 10 (GraphPad, USA). Data are either presented as mean with 95% confidence interval (CI), as percentage mean with 95% CI or as mean ± standard deviation (sdev) if not stated otherwise. The normality of the data, derived from independently obtained measurements or paired datasets, was analyzed and determined using the D’ Agostino Pearson omnibus normality test. An F‐test to compare variances was performed for each data set, and corrections regarding equal or unequal variances of data were considered for every subsequent statistical test. Statistical significance of the data was delineated using either a paired test for repeated measurements or an unpaired t‐test for data with similar variances as well as sample size, and an unpaired *t*‐test with Welch's correction was applied for data with unequal variances or unequal sample size. Connected data sets that did not pass normal distribution testing were tested for significant differences, applying the Mann Whitney‐U test and Wilcoxon matched pairs test. For multiple comparisons, either a one‐way ANOVA or a two‐way ANOVA with post‐hoc analysis was used for data showing Gaussian distribution. Data sets that did not show a normal distribution were analyzed using non‐parametric tests, ether the Kruskal‐Wallis with Dunn's post‐hoc analysis or Friedman test to examine any significant co‐dependencies. All presented analysis units (n) in the respective figures and figure legends refer to the number of biological replicates (independent observations) unless otherwise stated.

### Image and Data Processing

Presented histological images were brightness adjusted using Microsoft PowerPoint. Data analysis and calculations were performed in Microsoft Excel, and respective graphs were generated via GraphPad Prism 9 and 10. Statistical analysis was performed using GraphPad Prism 9 and 10. The cell counter plugin of FIJI/ImageJ was used to manually quantify Yap1 positive and negative cells of the immuno‐stained histological sections of synovial biochip organoids and synovial tissues.

### Ethics

The use of postoperative human tissue specimens collected with informed patient consent was approved by the ethics committee of the Medical University of Vienna (ethics permission numbers 1822/2017 and 1034/2020). The local ethical committee from the Austrian Federal Ministry of Education, Science and Research approved the primary study on transgenic mice (BMWFW‐66.009/0118‐II/3b/2013) from which the histological images of murine synovia sections were made. The equine tissue patient specimens were donated with informed owner consent.

### Ethics Approval Statement/Patient Consent Statement

The study was conducted in compliance with ethical standards and received approval from the ethics committee of the Medical University of Vienna (approval numbers: 1822/2017 and 1034/2020). Informed consent was obtained from all patients donating postoperative tissue specimens. For the histological images of murine synovia, ethical approval was granted by the Austrian Federal Ministry of Education, Science and Research (BMWFW‐66.009/0118‐II/3b/2013). Equine tissue specimens were obtained with informed owner consent.

## Conflict of Interest

The authors declare no conflict of interest.

## Author Contributions

All contributing authors are designated according to their respective contributions: E.I.R., F.J., W.H., S.T., P.E., H.P.K., and M.R. performed the conception and design of the study, E.I.R., A.S., M.G.V.M., M.M.S., M.C., R.G.‐B., B.R.‐M., J.A., R.L., I.G., and M.R. performed the acquisition of data, E.I.R., A.S., M.G.V.M., M.C., R.G.‐B., B.R.‐M., and M.R. performed analysis and the interpretation of data, J.A., R.L., I.G., W.H., S.T., R.W., and M.R. performed provision of study materials or patients, E.I.R. and M.R. drafted the article, E.I.R., A.S., M.G.V.M., M.M.S., M.C. R.G.‐B., B.R.‐M., J.A., R.L., I.G., F.J., W.H., S.T., P.E., H.P.K., R.W., and M.R. revised the article, E.I.R., A.S., M.G.V.M., M.M.S., M.C., R.G.‐B., B.R.‐M., J.A., R.L., I.G., F.J., W.H., S.T., P.E., H.P.K., R.W., and M.R. provided intellectual content, E.I.R., M.M.S., S.T., P.E., and M.R. provided statistical expertise., P.E., H.P.K., and M.R. obtained the funding, A.S., M.G.V.M., M.C., R.G.‐B., B.R.‐M., and M.R. provided administrative, technical, or logistic support, E.I.R., A.S., M.G.V.M., M.C., R.G.‐B., B.R.‐M., and M.R. performed collection and assembly of data, and E.I.R., A.S., M.G.V.M., M.C., R.G.‐B., B.R.‐M., J.A., R.L., I.G., F.J., P.E., H.P.K., R.W., and M.R. given final approval of the version to be submitted. M.R. ensures that all authors have seen and approved the manuscript and meet the criteria for authorship. M.R. and E.I.R. take responsibility for the manuscript content and contributed equally.

## Supporting information



Supporting Information

## Data Availability

The data that support the findings of this study are available from the corresponding author upon reasonable request.
