# Supplementary material for: An Animal‐Free Patient‐Derived Tissue‐Mimetic Biochip Model of the Human Synovial Membrane for Human‐Relevant Osteoarthritis Research
Source: Adv Healthc Mater. 2025 Jun 29;14(23):2404799. doi: 10.1002/adhm.202404799 (PMC12417762; doi:10.1002/adhm.202404799)
Supplement: Supplementary file 1 — Supporting Information [file ADHM-14-0-s001.docx]

Supporting Information - An Animal-Free Patient-Derived Tissue-Mimetic Biochip Model of the Human Synovial Membrane for Human-Relevant Osteoarthritis Research

**Eva I. Reihs^1,x^, Alexander Stoegner, Mateo G. Vasconez Martínez, Markus M. Schreiner, Melanie Cezanne, Ruth Gruebl-Barabas, Bettina Rodriguez-Molina, Juergen Alphonsus, Silvia Hayer, Richard Lass, Iris Gerner, Florien Jenner, Wolfgang Holnthoner, Stefan Toegel, Peter Ertl, Hans P. Kiener, Reinhard Windhager, and Mario Rothbauer*^1,x^**

**Content:** Figure SI 1-10; SI Table 1; Methods SI


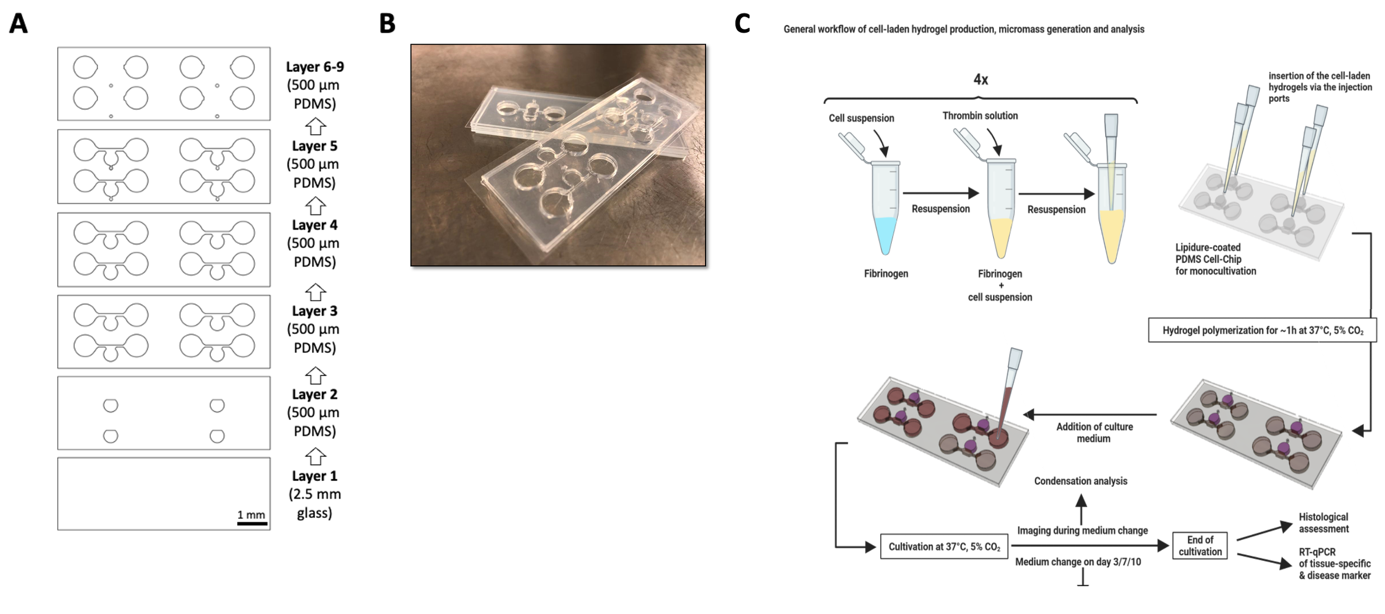


**Figure SI 1**. **(A)** Technical drawings of the individual 500 µm layers of the lay-by-layer prototyping approach for 26x76 mm object slide format. **(B)** Image of sterilized synovial organoid biochip prototypes. **(C)** Optimized protocol for the generation, maturation and analysis of non-animal synovial organoids of OA FLS origins.


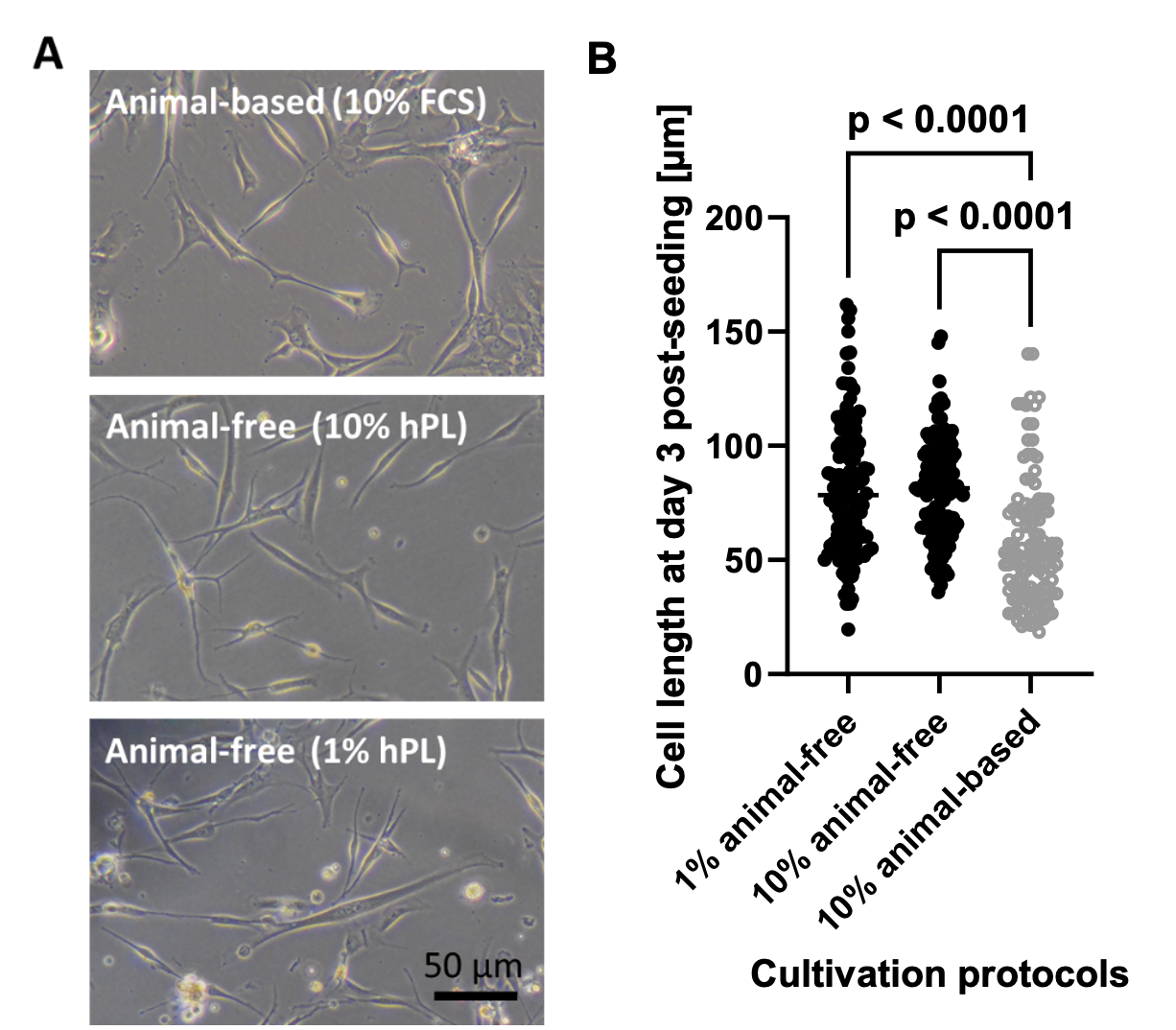


**Figure SI 2**. Fibroblast morphology on 2D surfaces with varying growth supplement conditions. Cellular elongation under the supplementation of 1 and 10% hpl and 10% FCS was monitored **(A)** and **(B)** analysed as µm cell length on day 3 after cell-seeding (n=65 cells for two individual donors). Data are expressed as mean ± sdev with Kruskal Wallis test with multiple comparisons.


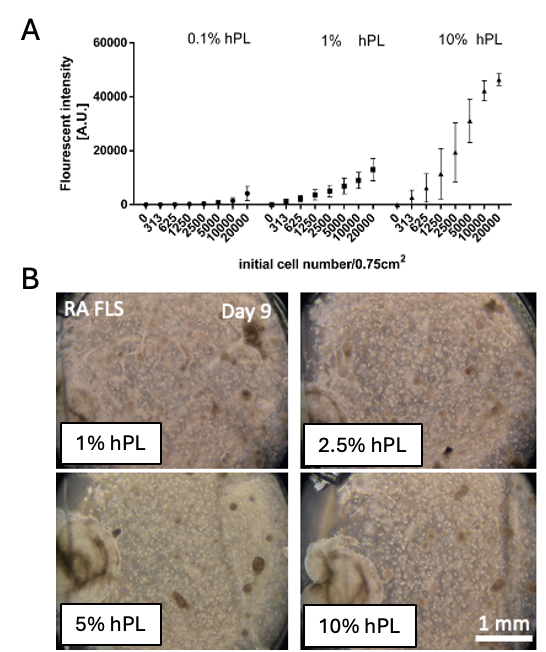


**Figure SI 3**. **(A)** Esterase activity analysis by PrestoBlue assay of OA patient-derived monolayer cultures of FLS when maintained in increasing concentrations of hPL medium supplement (n= 11). Data is shown as mean ± sdev. **(B)** Brightfield images of animal-derived RA FLS organoids in passages 4-5 when treated with increasing concentrations of hPL.


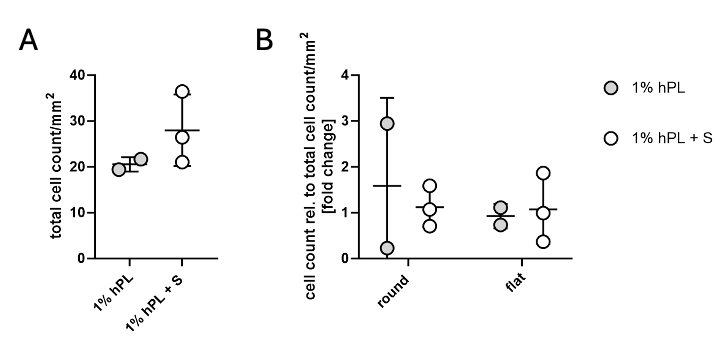


**Figure SI 4.** Impact of ascorbic acid supplementation on **(A)** organoid cellularity and, **(B)** cell morphology of non-animal synovial organoids (n=2-3) after 14 days of maturation. Data are shown as mean ± sdev.


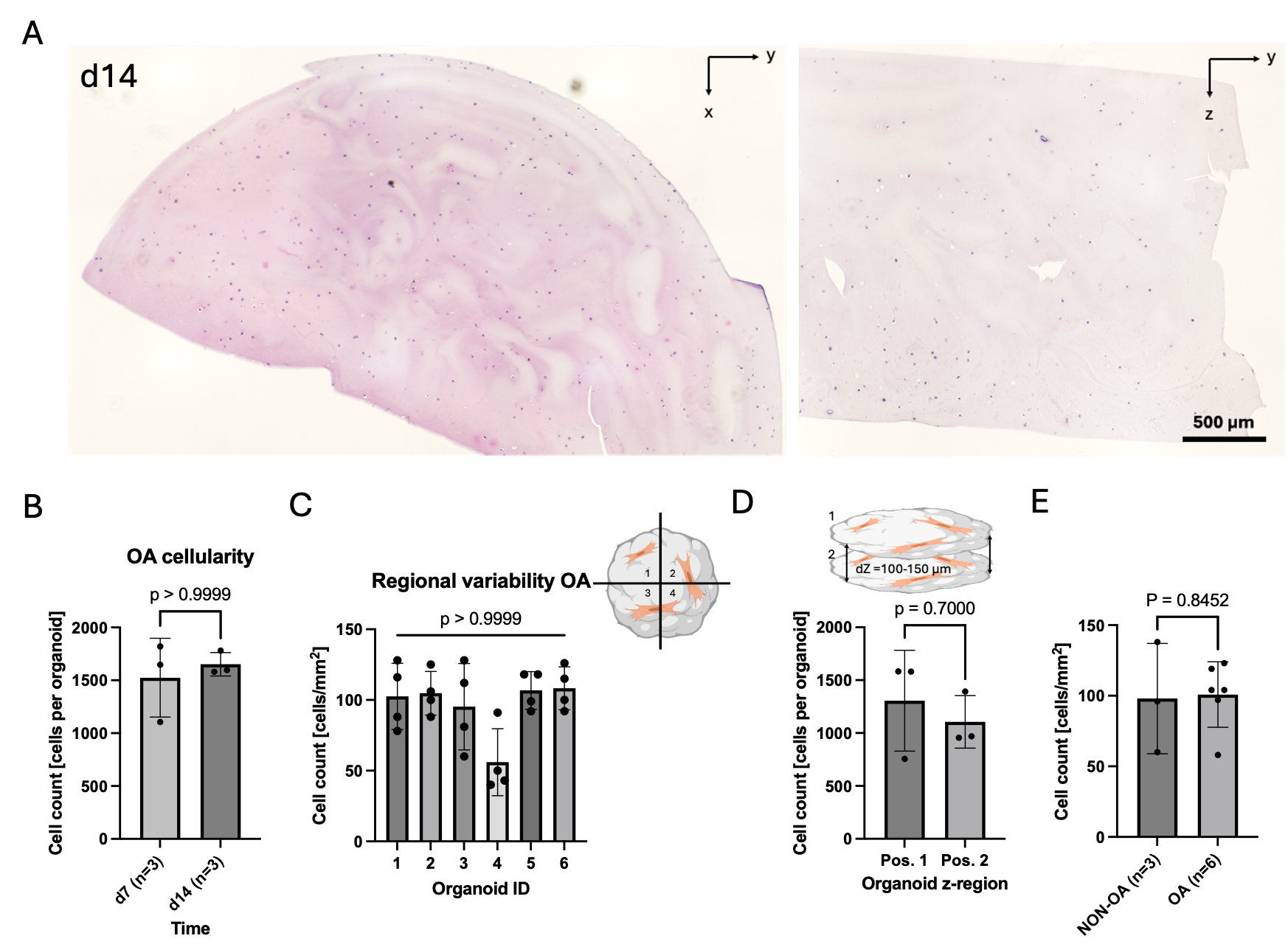


**Figure SI 5. Structural evaluation of synovial biochip organoids.** **(A)** Brightfield microscopy images of H&E-stained sections of synovial organoids at day 14 generated from human OA FLS under optimized animal-free conditions. The left image shows a transverse section across the organoid’s diameter, while the right panel displays a longitudinal section along the main axis of the organoid. **(B)** Quantitative comparison of cellular density at day 7 and day 14 (n = 3 biological samples), based on ImageJ cell counting per mm² from H&E sections. **(C)** Regional cell count variability analysis of six CMFDA-stained organoids at day 14 (n = 6 biological samples; Data are presented as mean ± sdev for four sectors per organoid tested with one-way ANOVA and Kruskal-Wallis with Dunn’s multiple comparisons test). **(D)** Regional variation in cellular distribution by z-section image analysis of two positions of CMFDA-stained organoids at day 14 (n = 3 biological samples; Data are presented as mean ± sdev with one-way ANOVA with Kruskal-Wallis with Dunn’s multiple comparisons test). **(E)** Comparative cell density analysis between OA-derived and commercial non-OA (healthy donor) FLS-derived organoids on day 14 (n = 6 for OA, n = 3 for non-OA). Quantification was performed using FIJI/ImageJ. Data are presented as mean ± sdev with the two-tailed Mann-Whitney test for (B), (D) and (E).

**
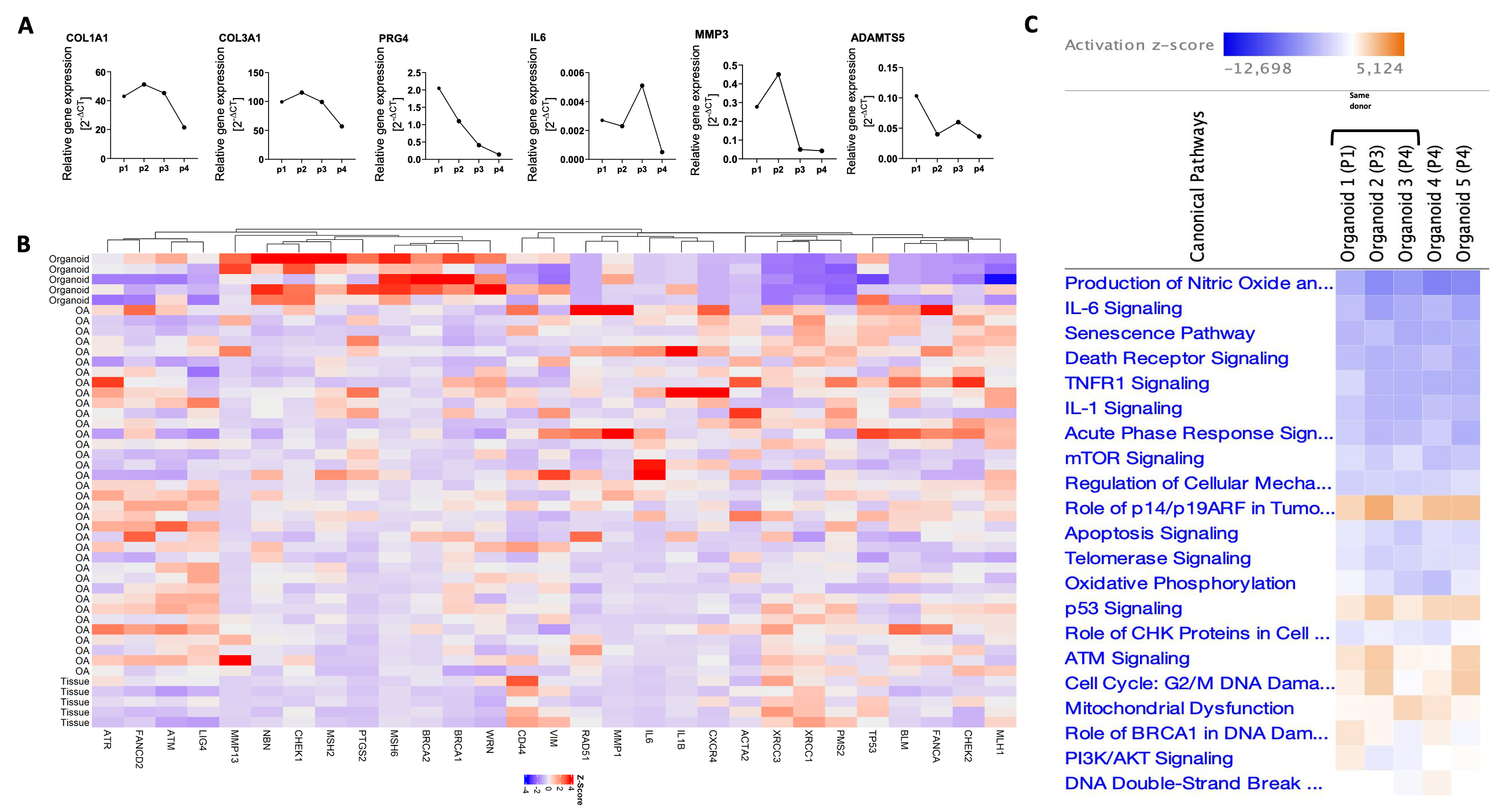
**

**Figure SI 6.** RT-qPCR and sequencing analysis of a synovial organoid cultures generated from OA synovial fibroblasts of serial passages. **(A)** Relative mRNA expression of patient-matched n=1 synovial organoids after day 14 post-seeding, followed over 4 increasing passages for COL1A1, COL3A1, PRG4, IL6, MMP3 and ADAMTS5. Expression values are shown as 2^-ΔCT mean value of n =2 technical analyses relative to SDHA. **(B)** RNA-seq analysis and **(C)** IPA™ pathway analysis of untreated FLS-derived patient organoids at day 14 post-seeding relative to open access sequencing data non-OA and OA-origin patient tissue data (GEO accession number GSE283079). Organoids 1-3 were generated from an identical donor up to passage number p4, while organoids 4-5 were two additional donors on P4.


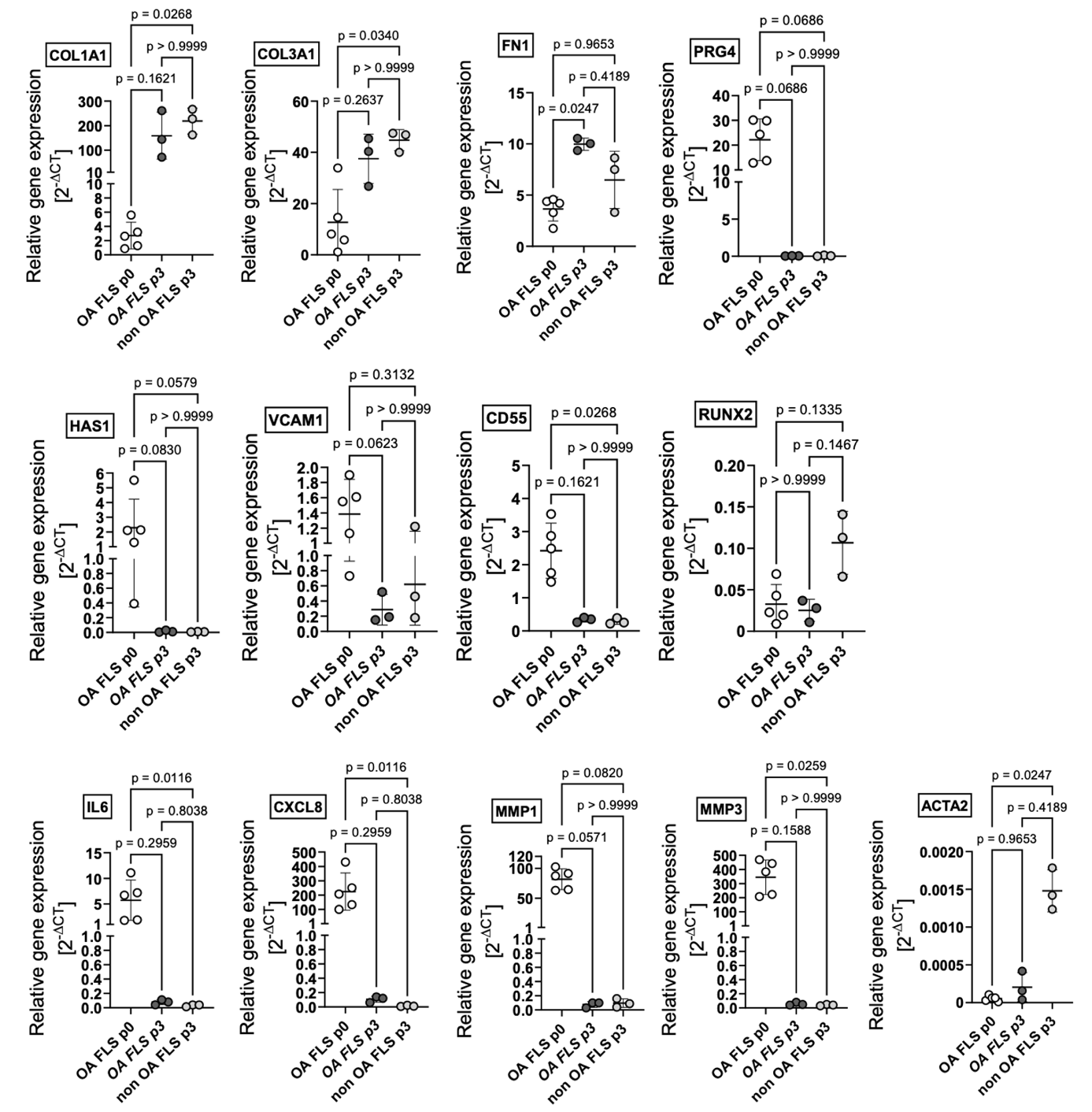


**Figure SI 7. Comparative RT-qPCR analysis of synovial organoid and FLS cultures.** Relative mRNA expression of patient-derived synovial FLS monolayers and organoids, as well as commercial non-OA FLS after day 7 post-seeding for COL1A1, COL3A1, FN1, PRG4, HAS1, VCAM1, CD55, RUNX2, IL6, CXCL8, MMP1, MMP3 and ACTA2. Expression values are shown as 2^-ΔCT mean value relative to SDHA of n =3-5 biological replicates.


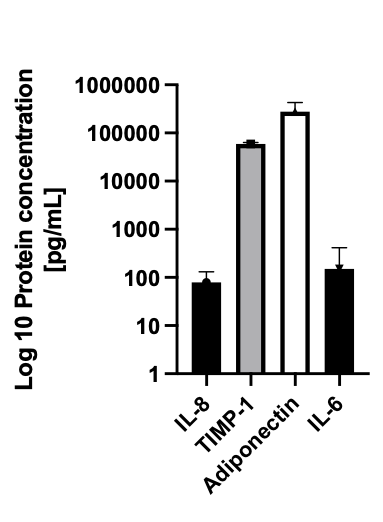


**Figure SI 8.** Cytokine Luminex analysis of patient synovial fluids of OA patients (KL3-4; n=24). Data are shown as mean ± sdev.


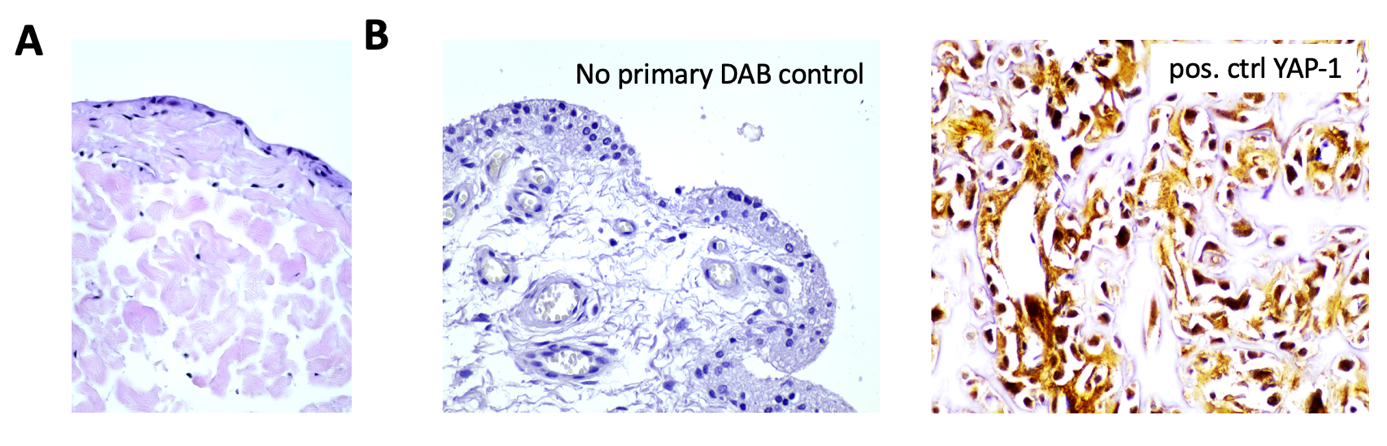


**Figure SI 9. (A)** Brightfield microscopy image of H&E-stained healthy equine synovial architecture of knee joint derived tissue specimens (400x magnification) **(B)** DAB^-^control (no primary Yap1 antibody) and Yap1^+^control of immune-stained human osteosarcoma (OSA) tissue.


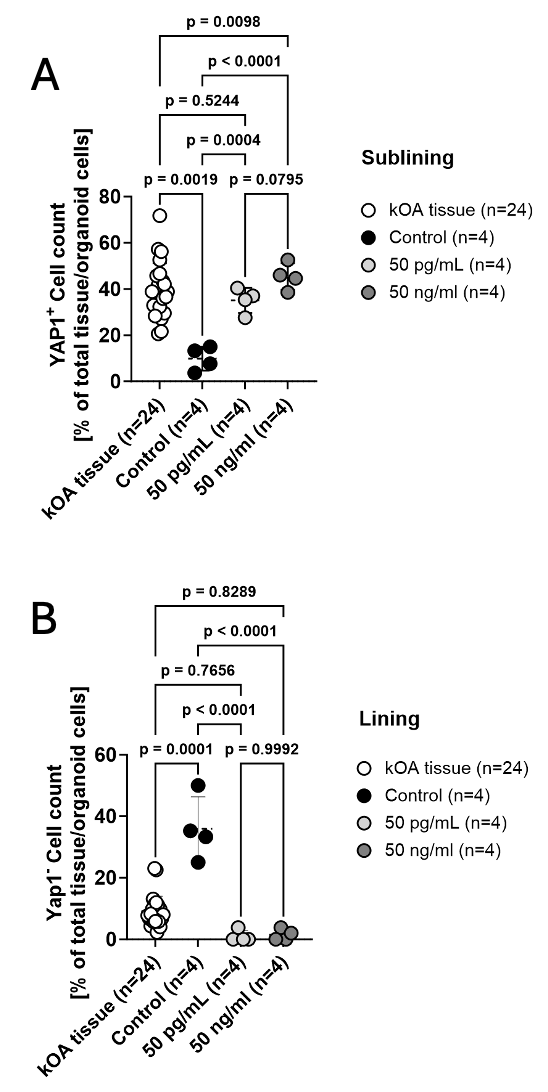


**Figure SI 10.** Microscopic analysis of either Yap1+ cell distribution in (**A**) sublining and Yap- cell population in (**B**) lining of kOA synovial patient tissue (n=24), untreated control biochips and micromasses that were stimulated with a low (50 pg/mL) and high (50 ng/mL) dose of a proinflammatory cytokine cocktail (n=4 for each group) Data are shown as mean with 95% CI.

**Table SI 1.** Calculated RSD % deriving from variable hPL concentrations and patient heterogeneity.

| **Cell count** | **0.1%** | **1%** | **10%** |
| --- | --- | --- | --- |
| **20000** | 64 | 32 | 5 |
| **10000** | 86 | 33 | 9 |
| **5000** | 73 | 42 | 26 |
| **2500** | 71 | 42 | 57 |
| **1250** | 75 | 52 | 82 |
| **625** | 69 | 56 | 85 |
| **313** | 0 | 53 | 96 |

**SI Methods:**

*RNA sequencing and analysis.* Total RNA libraries were prepared from high quality RNA (RIN >9) using the QuantSeq FWD protocol (Lexogen) with 19 PCR cycles and sequenced on a NextSeq500 (Illumina) in 1x75bp mode. Libraries were quality-checked using Bioanalyzer 2100 (Agilent) and Qubit dsDNA HS Assay (Invitrogen). Reads were processed with cutadapt v2.8^[1]^ and aligned to the GRCh38 genome^[2]^ with Gencode v29 annotations (Human genome annotations downloaded from Gencode(2018-11-22): ftp://ftp.ebi.ac.uk/pub/databases/gencode/Gencode_human/release_29/ gencode.v29.chr_patch_hapl_scaff.annotation.gtf.gz) using STAR v2.6.1a ^[3]^. Differential gene expression was analyzed with DESeq2 v1.46.0 ^[4]^. Ingenuity Pathway Analysis (IPA, QIAGEN) was conducted (Cutoffs organoids: -1.8/+1.8, Cutoffs tissue: -0,24, 0.24) focusing on DNA repair (HR, NHEJ, NER, MMR, BRCA1, ATM/ATR Signaling), cell cycle regulation (G1/S, G2/M, CHK proteins, p^[1]^53, p21 pathways), oxidative stress (NRF2, hypoxia, ROS production, glutathione, mitochondrial dysfunction, superoxide degradation), senescence (telomerase signaling, senescence, p14/p19ARF), and inflammatory/apoptotic signaling (IL-6, IL-1, NF-κB, TNFR1, Acute Phase Response, apoptosis, autophagy, death receptor, mTOR, PI3K/AKT) using the following filter sets:

„DNA Double-Strand Break Repair by Homologous Recombination, DNA Double-Strand Break Repair by Non-Homologous End Joining, Nucleotide Excision Repair (NER) Pathway, Mismatch Repair (MMR) Pathway, Role of BRCA1 in DNA Damage Response, ATM Signaling, ATR Signaling, Cell Cycle: G1/S Checkpoint Regulation, Cell Cycle: G2/M DNA Damage Checkpoint Regulation, Role of CHK Proteins in Cell Cycle Checkpoint Control, p53 Signaling, p21 (CDKN1A) Signaling, NRF2-mediated Oxidative Stress Response, Hypoxia Signaling in the Cardiovascular System, Production of Nitric Oxide and Reactive Oxygen Species in Macrophages, Oxidative Phosphorylation, Mitochondrial Dysfunction, Glutathione Redox Reactions I, Superoxide Radicals Degradation, Senescence Pathway, Telomerase Signaling, Role of p14/p19ARF in Tumor Suppression, Regulation of Cellular Mechanics by Calpain Protease, IL-6 Signaling, IL-1 Signaling, NF-κB Signaling, TNFR1 Signaling, Acute Phase Response Signaling, Apoptosis Signaling, Autophagy Pathway, Death Receptor Signaling, mTOR Signaling, PI3K/AKT Signaling“.

*Fluorescence imaging.* Live-cell staining of FLS-containing organoids was performed with fluorescent cell tracker dye (Green CMFDA, Thermo Fisher) before pelleting and hydrogel mixing according to the manufacturer's staining protocol (5 µM and 45 min incubation with 2 washing steps) using an Eclipse Ti2-E setup with AX confocal module. FIJI Imagej was used for image processing (thresholding, area measure and particle count function).

**Bibliography**

[1] M. Martin, *EMBnet.journal* **2013**, *17*, 9.

[2] NCBI, “Genome assembly GRCh38,” can be found under https://www.ncbi.nlm.nih.gov/datasets/genome/GCF_000001405.26/, **n.d.**

[3] A. Dobin, C. A. Davis, F. Schlesinger, J. Drenkow, C. Zaleski, S. Jha, P. Batut, M. Chaisson, T. R. Gingeras, *Bioinformatics* **2013**, *29*, 15.

[4] M. I. Love, W. Huber, S. Anders, *Genome Biol.* **2014**, *15*, 1.
